# Glomus cell heterogeneity underpins distinct carotid body chemoreflex pathways: implications for hypertension

**DOI:** 10.1093/cvr/cvag118

**Published:** 2026-05-24

**Authors:** Xin Shen, Audrys G Pauza, Olivia M S Gold, Igor S A Felippe, Pratik Thakkar, Julian F R Paton

**Affiliations:** Department of Physiology, Faculty of Medical & Health Sciences, Manaaki Mānawa–The Centre for Heart Research, University of Auckland, Auckland, New Zealand; Department of Physiology, Faculty of Medical & Health Sciences, Manaaki Mānawa–The Centre for Heart Research, University of Auckland, Auckland, New Zealand; Department of Physiology, Faculty of Medical & Health Sciences, Manaaki Mānawa–The Centre for Heart Research, University of Auckland, Auckland, New Zealand; Department of Physiology, Faculty of Medical & Health Sciences, Manaaki Mānawa–The Centre for Heart Research, University of Auckland, Auckland, New Zealand; Department of Physiology, Faculty of Medical & Health Sciences, Manaaki Mānawa–The Centre for Heart Research, University of Auckland, Auckland, New Zealand; Department of Physiology, Faculty of Medical & Health Sciences, Manaaki Mānawa–The Centre for Heart Research, University of Auckland, Auckland, New Zealand

**Keywords:** Peripheral chemoreflex, Carotid body, Hypertension, Glomus cell signalling, Sympathetic nerve activity

## Abstract

**Aims:**

The carotid body (CB) is a multimodal chemosensory organ, yet how it encodes differential external stimuli to drive distinct peripheral chemoreflex responses remains unclear. This study aimed to define differences in intrinsic cellular mechanisms underlying CB signalling and to investigate how these processes are altered in pre-hypertension.

**Methods and results:**

We used potassium cyanide (KCN) as a hypoxia mimetic to activate the CB.

K^+^ channels implicated in hypoxia sensing were pharmacologically inhibited, with TWIK-related acid-sensitive K^+^ (TASK) channels targeted using Ba^2+^ or ML365 and BK/Kv channels targeted using TEA + 4AP or iberiotoxin. We combined *in vitro* Ca^2+^ imaging of dissociated glomus cells, *ex vivo* carotid sinus nerve (CSN) recordings, and an *in situ* double-perfused working heart-brainstem preparation to investigate chemosensory signalling in juvenile Wistar and age-matched pre-hypertensive spontaneously hypertensive rats (SHRs). KCN application to the CB *ex vivo* evoked a biphasic CSN response, consisting of two temporally distinct components. This biphasic response was exaggerated in SHRs, with disproportionate changes in the magnitude of each component. Ca^2+^ imaging revealed that these components reflect distinct glomus cell subpopulations, characterized by transient vs. prolonged KCN-evoked Ca^2+^ events, with their relative proportions shifted in hypertension. Pharmacological dissection suggested that differential inactivation of K^+^ channels contributes to these divergent Ca^2+^ dynamics. *In situ*, we confirmed that localized Ba^2+^ applied to the CB facilitated stronger tachypnoeic and bradycardic chemoreflex evoked responses than TEA + 4AP, consistent with functional heterogeneity among glomus cell populations. Compared with Wistar rats, *in vitro* TASK and BK/Kv channel expression and activity were differentially altered in SHRs, accompanied by corresponding changes in *in situ* chemoreflex characteristics.

**Conclusion:**

We identify, for the first time, distinct glomus cell subpopulations defined by their K^+^ channel expression that differentially contribute to CB signalling patterns and distinct chemoreflex pathways. These findings provide mechanistic support for the recently proposed ‘ribbon cable’ hypothesis, whereby discrete glomus cell populations couple to specific chemoreflex outputs. These findings offer new insight into CB signalling complexity and its role in autonomic dysfunction during the early stages of hypertension.

## Graphical Abstract

**Graphical Abstract:**
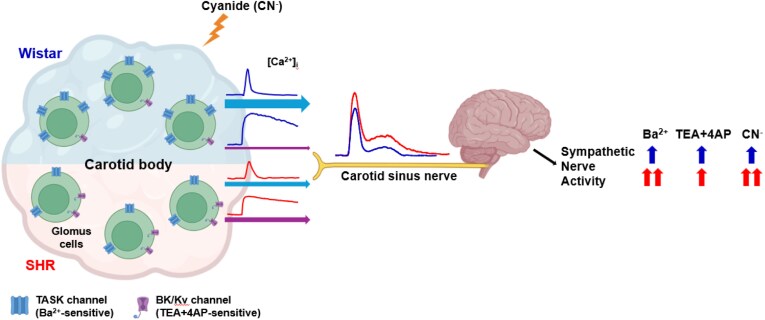

**Time of primary review: 37 days**

## 1. Introduction

Hypertension is often labelled the silent killer because of its asymptomatic nature and is a leading cause of premature death worldwide. Globally, the treatment rate was 47% (43–51) among women and 38% (35–41) among men. However, fewer than half of treated individuals achieved hypertension control, yielding overall control rates of 23% (20–27) in women and 18% (16–21) in men.^1^ Epidemiological studies show that even among patients who are considered ‘treated’ for high blood pressure, a significant number still experience strokes and heart attacks due to poorly controlled blood pressure surges.^2^

Although the pathophysiology of hypertension is complex, it is well recognized that impaired autonomic cardiovascular control leads to aberrant sympathetic activation of the heart and peripheral vessels, promoting haemodynamic dysfunction, inflammation, and end-organ damage.^3,4^ Our group and others have demonstrated that one of the major drivers of excessive sympathetic nerve activity in hypertension originates from carotid body (CB) dysfunction.^5–7^ Located bilaterally at the bifurcation of the common carotid arteries, the CB is a key sensor of blood oxygen, carbon dioxide, and pH. In response to hypoxia, hypercapnia, or acidosis, it increases carotid sinus nerve (CSN) discharge, activating neurons in the nucleus tractus solitarius, the primary integrative centre in the medulla, enhancing sympathetic output and triggering a host of chemoreflex responses collectively resulting in acute elevations in blood pressure.^8^ In disease, the CB displays elevated basal activity and exaggerated responses to stimuli.^9^ While CB denervation^6,10^ or resection^7^ can attenuate sympathetic output and blood pressure, these approaches are irreversible and clinically untenable. As a result, attention has focused on identifying novel pharmacological targets within the CB in the fight against cardiometabolic disease.

The CB contains glomus, or type I, cells that sense hypoxia and stimulate petrosal afferent terminals to drive chemoreflex responses, and the basic chemo-transduction mechanisms underlying this process are known (for reviews, see the work by Shen and Paton^11^ and the work by Iturriaga et al.^12^). In short, when glomus cells become stimulated in conditions of hypoxia, they depolarize. This results in an increase in intracellular Ca^2+^, which subsequently drives the release of different neurotransmitters, including dopamine, norepinephrine, nitric oxide, serotonin, neuropeptides, adenosine, and ATP onto the CSN afferent terminals.^7,13–18^ Work over the years has identified that this process crucially depends on the inhibition of mitochondrial respiration^19–22^ in glomus cells and the subsequent inactivation of key K^+^ channels, including voltage-gated K^+^ channels, large conductance BK channels and TWIK-related acid-sensitive K^+^ (TASK) channels.^12,23–25^ What remains unclear is why multiple K^+^ channels exist and whether they are present on all glomus cells or on different sub-populations. It has been proposed that distinct morphological^26,27^ and functional^28–30^ differences among glomus cell populations may dictate their differential sensitivity to hypoxia. Therefore, the possibility remains that distinct sub-populations of glomus cells defined by their expression of different K^+^ channels may modulate different populations of petrosal afferents mediating individual components of the chemoreflex responses, such as the hyperventilation vs. hypertension.

In this study, we comprehensively characterize the functional role of specific K^+^ channels within glomus cells for mediating the biphasic CSN response evoked by potassium cyanide (KCN), a potent and widely used hypoxia mimetic.^5–7^ We show that this biphasic response is (1) mediated by different glomus cell subpopulations with distinct K^+^ channel expression, leading to different chemoreflex responses, and (2) the expression and activity of these K^+^ channels are differentially altered in glomus cells from spontaneous hypertensive rats. These findings reveal the functional meaning of glomus cell heterogeneity and may have implications for aberrant chemoafferent signalling in hypertension.

## 2. Methods

### 2.1 Animal use and ethical approval

A total of 40 juvenile (4–6 weeks) Wistar rats (37 males and 3 females; Crl:WI outbred; Charles Rivers) and 19 Spontaneously Hypertensive (male, SHR/NHsd; Envigo) rats were used for this study. All rats were obtained from the Vernon Jansen Unit (VJU, University of Auckland, New Zealand). Animals were housed in conventional Tecniplast 1500 rat cages with controlled temperature (21 ± 2°C), humidity (55% ± 10%), and 12 h light–dark cycle. Animals had unlimited access to food and water (*ad libidum*). Following transportation, animals were allowed to acclimatize to the housing facility for at least 7 days prior to any experimental procedures. Animals were randomly allocated to each experimental group. Given the highly technical nature of the studies and pronounced differences in basal physiological parameters between the strains, it was not possible to blind them. All procedures were approved by the University of Auckland Animal Ethics Committee (AEC 26798) and carried out at the University of Auckland, New Zealand, with strict adherence to local rules and regulations. These procedures also conform to the guidelines from Directive 2010/63/EU of the European Parliament on the protection of animals used for scientific purposes or the NIH Guide for the Care and Use of Laboratory Animals.

## 3. Results

### 3.1 Hypoxia mimetic evokes biphasic carotid sinus nerve response from the carotid body and is potentiated in the SHR

**Figure 1:**
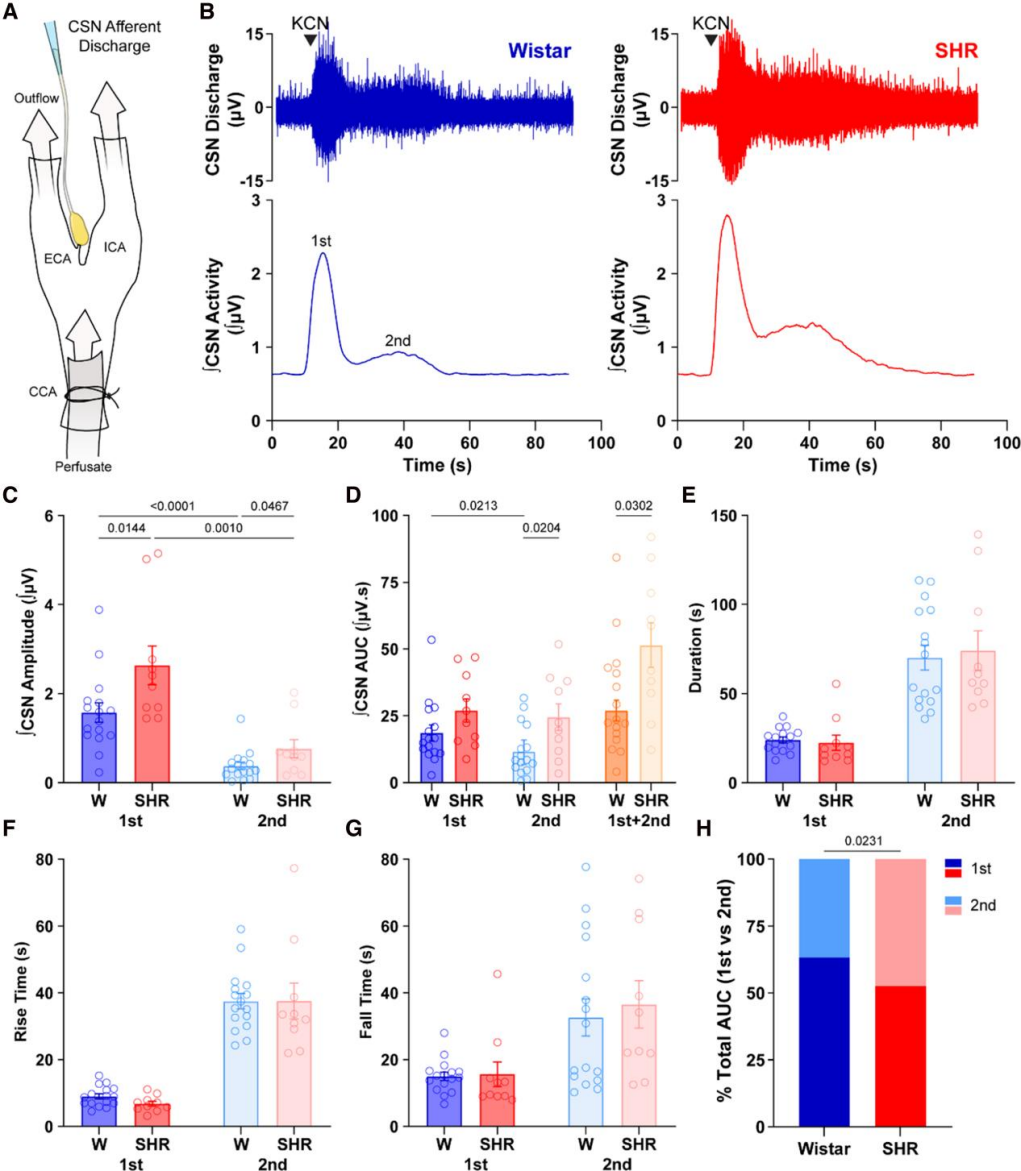
A biphasic CSN afferent discharge is evoked by the addition of KCN to the CB, and is augmented in the SHR. (*A*) Schematic showing how CSN afferent discharge is recorded in the *ex vivo* CB-CSN preparation (CSN, carotid sinus nerve; ECA, external carotid artery; ICA, internal carotid artery; CCA, common carotid artery). (*B*) Representative traces showing CSN discharge (top) and integrated CSN activity (bottom) in the Wistar and SHR following a bolus injection of KCN (0.08%, 100 µL). The resulting two responses are labelled 1st and 2nd, respectively. (*C*) Amplitude; (*D*) area under the curve; (*E*) duration; (*F*) rise time, and (*G*) fall time of the integrated biphasic response between the Wistar and the SHR. (*H*) 1st and 2nd component AUCs, expressed as a percentage of total AUC, between Wistar and SHR. For Wistar, *n* = 16 animals; SHR, *n* = 10 animals. Error bars are ± SEMs. *P* values calculated were using the Mann–Whitney *U* test.

We observed a distinct biphasic CSN response when potassium cyanide (KCN) was delivered arterially to stimulate the CB in the *ex vivo* CB-CSN preparation (*Figure 1A*).^5,31^ This biphasic response (hereafter described as the ‘1st’ and ‘2nd’ components) was present in both Wistars and SHRs (*Figure 1B*). First, we noted that the amplitude of the integrated CSN response was larger for the 1st than the 2nd components in both strains, and that both components were augmented in SHR compared with Wistar rats (*Figure 1C*). However, when we looked at the integrated area (area under the curve, AUC) as a measure of total CSN output, we found the 1st component was significantly larger than the 2nd only in Wistars, not SHRs (*Figure 1D*). For both 1st and 2nd components, SHRs tended to exhibit increased total CSN output when compared to Wistars, and when these components were summed, this difference was significant (*Figure 1D*). We found no differences in duration, rise time and fall time between Wistar and SHRs for either component (*Figure 1E–G*). We also investigated and found no strain difference in the time between peak amplitudes across the two components (see Supplementary material online, *Figure S5A*). Similarly, comparing the ratio of the 1st component amplitude to the 2nd showed no difference between Wistar and SHRs (see Supplementary material online, *Figure S5B*). On the other hand, the ratio of AUC of the 1st component to the 2nd component was significantly smaller in the SHR when compared to Wistar rats (*Figure 1H*). Taken together, applying KCN to the CB drives a unique biphasic CSN response that is potentiated in the SHR.

### 3.2 CB glomus cells exhibit two distinct types of Ca^2+^ events following KCN application

**Figure 2:**
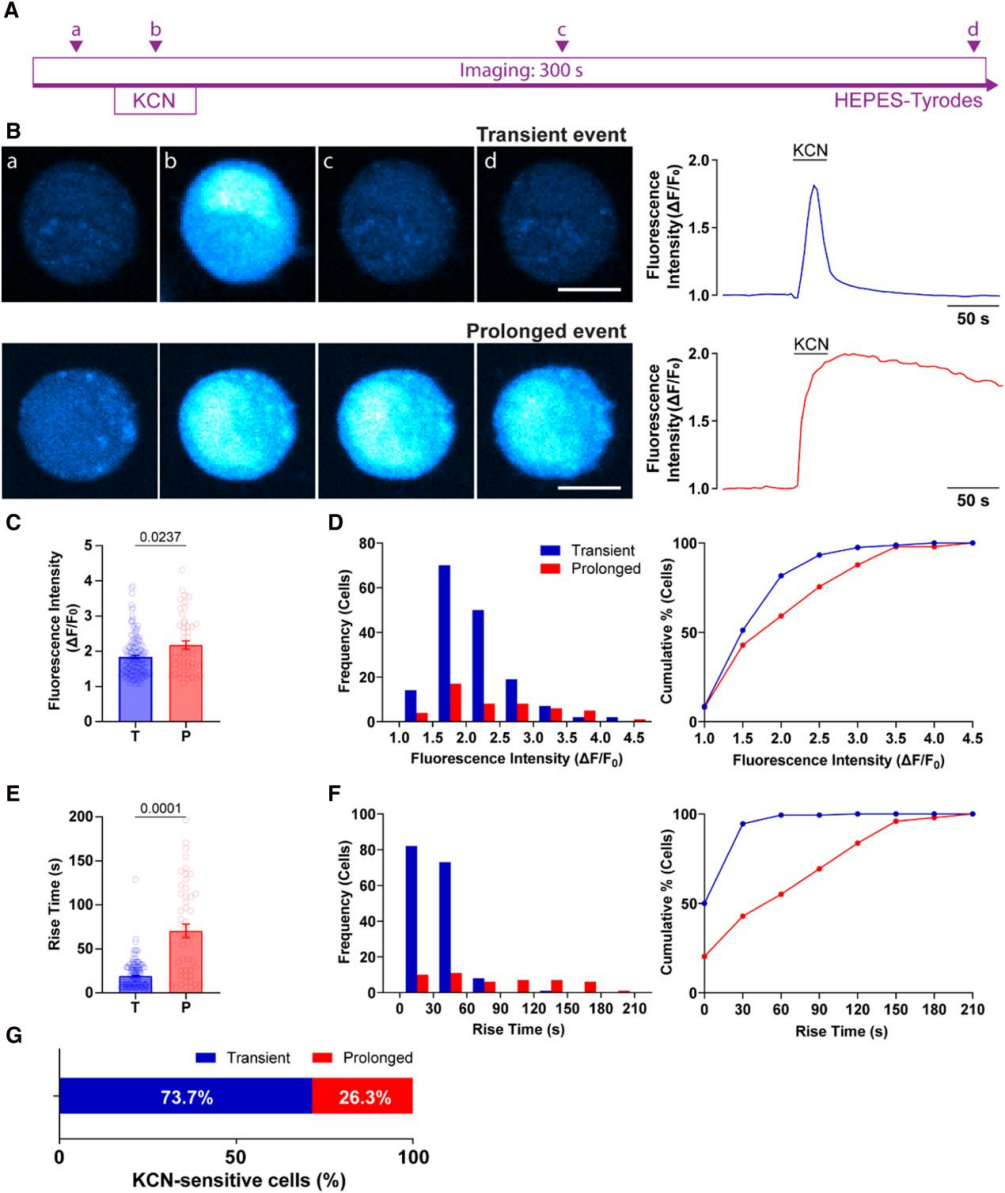
Wistar CB glomus cells exhibit two distinct types of Ca^2+^ events following KCN application. (*A*) Experimental protocol. Arrows (a–d) indicate where each of the representative images from (*B*) was taken. (*B*) Representative images (left) and traces (right) of a ‘transient’ (T) Ca^2+^ event (top) and a ‘prolonged’ (P) Ca^2+^ event (bottom) in two different glomus cells following the addition of KCN (1 mM). (*C*) Fluorescence intensity between T and P Ca^2+^ events. (*D*) Frequency and cumulative frequency distribution of fluorescence intensity of transient and prolonged Ca^2+^ events from all glomus cells. (*E*) Rise time between T and P Ca^2+^ events. (*F*) Frequency and cumulative frequency distribution of rise time of transient and prolonged Ca^2+^ events. (*G*) Percentage distribution of T and P Ca^2+^ events in all glomus cells that responded to KCN. *n* = 213 cells/6 animals. Error bars are ± SEMs. *P* values calculated were using the Mann–Whitney *U* test.

Given the differences in the CN-evoked biphasic CSN discharge, we assessed whether different subpopulations of glomus cells could contribute to this response. We administered KCN on dissociated, Fluo-4-loaded glomus cells from Wistar rats and quantified subsequent changes in [Ca^2+^]_i_ (*Figure 2A*).

Of the imaged cells (*n* = 213), two subpopulations were observed, each exhibiting a distinct type of Ca^2+^ event, either ‘transient’ or ‘prolonged’ (*Figure 2B*). On average, the transient event has a fluorescence intensity (Δ*F*/*F*_0_, mean ± SEM) of 1.83 ± 0.04 (*Figure 2C*). It is characterized by a rapid rise to peak intensity (*Figure 2E*), followed by a short decay back to baseline (see Supplementary material online, *Figure S6A*) and a mean duration of 57 ± 2.7 s (see Supplementary material online, *Figure S6B*). In contrast, the prolonged event is significantly larger in fluorescence intensity (*Figure 2C* and *D*) and slower in rise time (*Figure 2E* and *F*). It has a markedly slower fall time and did not recover to baseline during our image acquisition window of 5 min, and hence could not be fully quantified. As a result, the net influx of Ca^2+^ is significantly larger in magnitude for the prolonged response compared to the transient response (see Supplementary material online, *Figure S6C*). Finally, the frequency distribution of the two cell types illustrates a large majority of transient responses (∼74%; *Figure 1G*). Thus, at least two subpopulations of glomus cells were characterized based on their distinct [Ca^2+^]_i_ response to KCN.

### 3.3 SHR exhibits smaller KCN-mediated Ca^2+^ events but increased total Ca^2+^ influx due to increased proportion of cells exhibiting prolonged events

**Figure 3:**
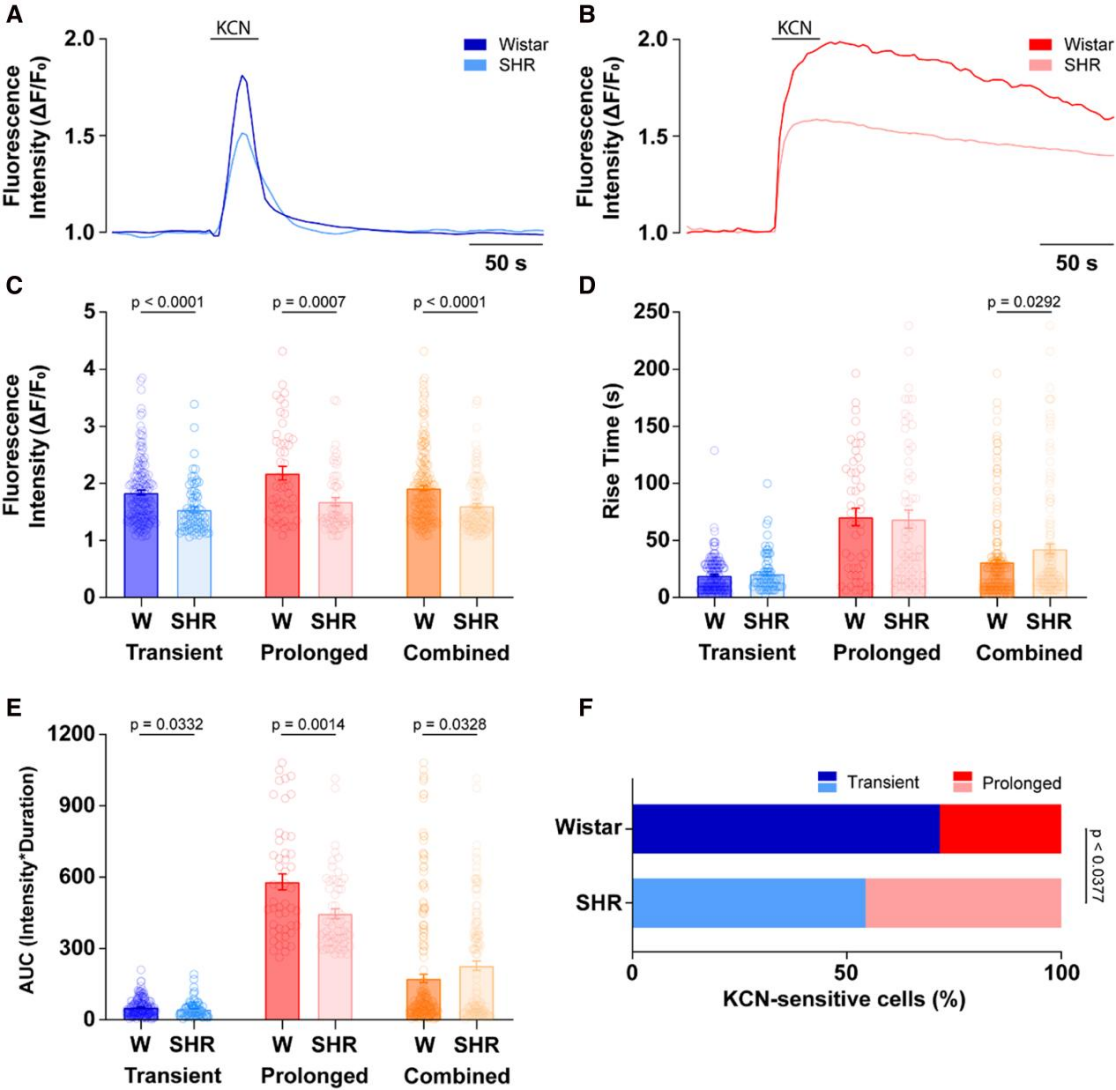
SHR exhibits smaller KCN-mediated Ca^2+^ events but increased total Ca^2+^ influx due to increased proportion of glomus cells displaying prolonged events. (*A*) Representative trace comparing the transient event between Wistars and SHRs. (*B*) Representative trace comparing the prolonged event between Wistars and SHRs. (*C*) Fluorescence intensity, (*D*) rise time, (*E*) AUC (Ca^2+^ influx magnitude) of transient, prolonged, and combined KCN-mediated Ca^2+^ events between Wistars and SHRs. (*F*) Relative distribution of transient and prolonged Ca^2+^ events in KCN-sensitive glomus cells from Wistars and SHRs. For Wistar, *n* = 213 cells/6 animals; for SHR, *n* = 136/9 animals. Error bars are ± SEMs. *P* values were calculated using the Mann–Whitney *U* test.

Both the transient and prolonged Ca^2+^ events evoked by KCN were present in the SHR (*Figure 3A, B*), but with significantly reduced fluorescence intensity (*Figure 3C*) and, hence, Ca^2+^ influx (*Figure 3E*). For both events, there were no differences in rise time (*Figure 3D*), fall time (see Supplementary material online, *Figure S7A*), duration (see Supplementary material online, *Figure S7B*), or event frequency (see Supplementary material online, *Figure S7C*) between Wistars and SHRs. When all Ca^2+^ events were combined, the fluorescence intensity of the SHR remained depressed when compared to the Wistar (*Figure 3C*). However, we observed a significant increase in the rise time (*Figure 3D*) and total Ca^2+^ influx (*Figure 3E*) in combined responses, that is, the average response across all cells in the SHR. Note that in both the Wistar and the SHR, prolonged events carried on longer than our imaging window (5 min), hence we could not directly calculate and compare the combined fall time and duration between the two strains. To consolidate these findings, we compared the overall proportion of KCN-sensitive glomus cells exhibiting transient and prolonged events between Wistars and SHRs and identified a significant shift towards having more cells with prolonged events in the SHR (*Figure 3F*). This increase in the number of cells with a prolonged response is consistent with the AUC of the 2nd component (to the total CSN response) being larger in SHRs (47%) compared to Wistars (37%; *Figure 1H*). In summary, KCN-mediated Ca^2+^ events in single glomus cells are substantially smaller in magnitude in the SHR, yet the overall Ca^2+^ influx at the population level is increased due to a higher proportion of cells exhibiting prolonged events.

### 3.4 Activation of different K^+^ channels evokes distinct Ca^2+^ event types in glomus cells

**Figure 4:**
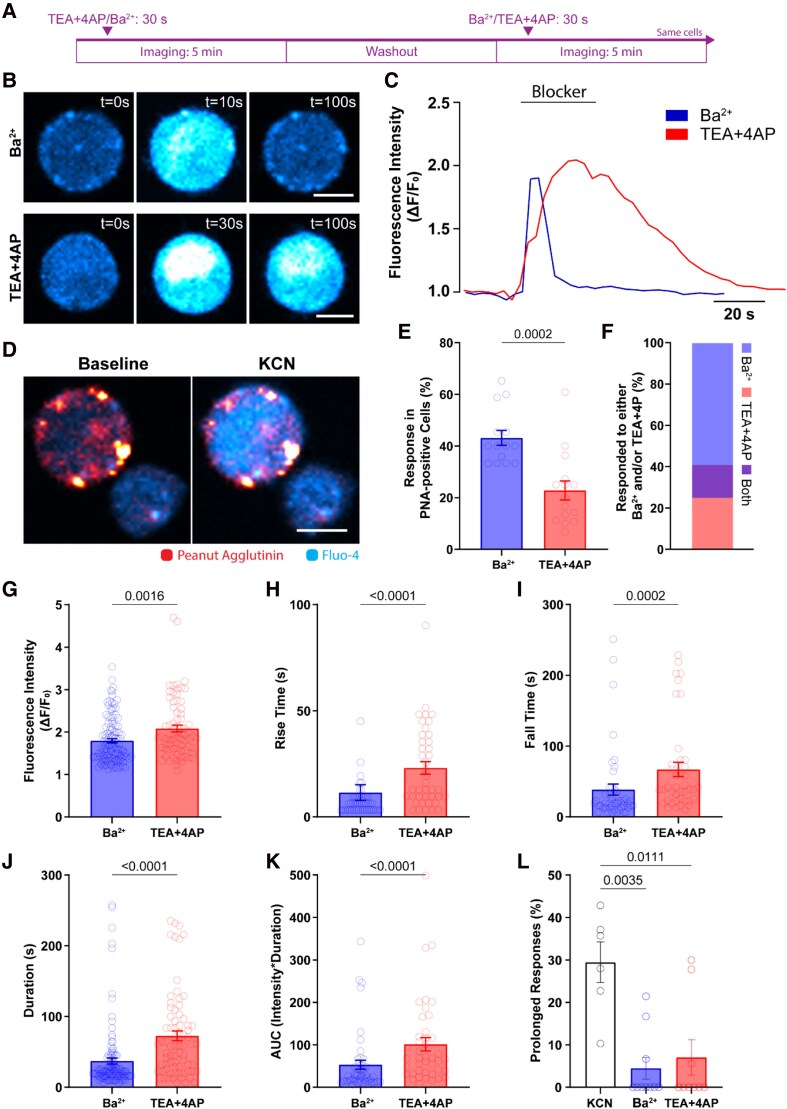
Activating different K^+^ channels evokes Ca^2+^ events with distinct profiles in Wistar CB glomus cells. (*A*) Experimental protocol outline depicting a single imaging run. TASK channel inhibitor Ba^2+^ (10 mM) or Kv/BK channel inhibitor TEA (10 mM) + 4AP (5 mM) were given in randomized orders in each run. Each run was carried out on either the same (to examine co-expression of K^+^ channels) or a different set of glomus cells between washouts. (*B*) Representative confocal time series of Fluo-4-loaded glomus cells responding to either Ba^2+^ or TEA + 4AP. Scale bar: 5 µm (*C*) Representative traces showing two distinct types of Ca^2+^ events evoked by the addition of either Ba^2+^ or TEA + 4AP in glomus cells. (*D*) Representative images showing that KCN (1 mM)-responsive glomus cells are also positive for peanut agglutinin (PNA) labelling. Scale bar: 5 µm. (*E*) Ba^2+^- or TEA + 4AP-sensitive glomus cells as a percentage of all PNA-positive cells. (*F*) Percentage of glomus cells that responded to Ba^2+^ and/or TEA + 4AP. Here, protocol was used on the same cells. (*G*–*K*) Ba^2+^- and TEA + 4AP-mediated Ca^2+^ event intensity, rise time, fall time, duration, and AUC (Ca^2+^ influx magnitude) in glomus cells. (*L*) Percentage of prolonged Ca^2+^ events evoked by KCN, Ba^2+^, or TEA + 4AP. For imaging runs performed on different glomus cells, *n* = 91 cells/5 animals and 71 cells/5 animals for Ba^2+^ and TEA + 4AP, respectively. For runs performed on the same cells, *n* = 69 cells/5 animals. Total, *n* = 231 cells/5 animals. Error bars are ± SEMs. *P* values were calculated using the Mann–Whitney *U* test.

We used either Ba^2+^ to inhibit TASK channels, which are insensitive to TEA and 4AP^32–34^ and, separately, a combination of TEA and 4AP to inhibit Kv/BK channels. In our hands (*Figure 4A*), both interventions evoked Ca^2+^ events (*Figure 4B*). Furthermore, we observed two distinct event profiles depending on which K^+^ channels were blocked. Specifically, a transient event was evoked in the presence of Ba^2+^ and a longer-lasting event with greater fluorescence intensity in the presence of a combination of TEA + 4AP (*Figure 4C*), reminiscent of the biphasic responses in the CSN and the transient and prolonged Ca^2+^ events in isolated glomus cells. Using peanut agglutinin (PNA) as a positive marker for glomus cells (*Figure 4D*),^35^ we calculated the relative proportion of Ba^2+^- and TEA + 4AP-sensitive glomus cells to be 43.2% (160 cells imaged) and 22.8% (140 cells imaged), respectively (*Figure 4E*), leaving a modest subset of cells (34%) insensitive to Ba^2+^ or TEA + 4AP.

When we further classified glomus cells that had responded to either Ba^2+^ and/or TEA + AP (*Figure 4F*), we observed three distinct subtypes of cells: cells sensitive exclusively to Ba^2+^ (41/69); cells sensitive exclusively to TEA + 4AP (17/69), and a proportion sensitive to both Ba^2+^ and TEA + AP (11/69). Comparing between subpopulations of glomus cells which had only responded to one intervention revealed that Ba^2+^ inhibition produced Ca^2+^ events that were significantly smaller in fluorescence intensity (*Figure 4G*), faster in rise (*Figure 4H*), and fall time (*Figure 4I*) and shorter in duration (*Figure 4J*), resulting in significantly lower total Ca^2+^ influx (*Figure 4K*) relative to TEA + AP. Due to striking similarities between KCN-mediated prolonged responses and TEA + 4AP-evoked Ca^2+^ events, we explored whether TEA + 4AP (or Ba^2+^) inhibition was sufficient to reliably reproduce the prolonged response. *Figure 4L* shows that while prolonged responses could be triggered by Ba^2+^ or TEA + 4AP inhibition in glomus cells, their rate of occurrence when compared to those evoked by KCN application was significantly lower.

Our initial pharmacological interrogation using the broad K^+^ channel blockers Ba^2+^ and TEA + 4AP revealed marked heterogeneity in glomus cell Ca^2+^ events. However, given the recognized lack of selectivity of these agents, we next repeated the protocol using the selective TASK channel inhibitor ML365^36^ and the BK channel blocker iberiotoxin (IbTx)^37^ to validate these findings. Out of 137 PNA-positive glomus cells, 28.3% were ML365-sensitive and 22.3% were IbTx-sensitive (see Supplementary material online, *Figure S8C*). These proportions were lower than those observed with Ba^2+^ or TEA + 4AP and were more evenly distributed across the population. Consistent with this, the relative distribution of event phenotypes was more balanced, with three subpopulations of cells responding exclusively to ML365 (20/50), exclusively to IbTx (14/50), or to both inhibitors (16/50) (see Supplementary material online, *Figure S8D*).

Analysis of Ca^2+^ event characteristics showed that selective blockade of TASK or BK channels largely recapitulated the effects observed with broad K^+^ channel inhibition (see Supplementary material online, *Figure S8B*). Compared with ML365-sensitive cells, IbTx-sensitive cells exhibited a trend towards higher fluorescence intensity and prolonged rise times (see Supplementary material online, *Figure S8G*, *H*). In addition, IbTx-sensitive cells displayed significantly greater fall time, event duration, and AUC compared with ML365-sensitive cells (see Supplementary material online, *Figure S8I*–*K*). In contrast to Ba^2+^ and TEA + 4AP, neither ML365 nor IbTx induced prolonged Ca^2+^ responses (see Supplementary material online, *Figure S8L*).

Finally, we explored whether there are differences in Ca^2+^ events between glomus cells expressing both Ba^2+^ and TEA + 4AP sensitivity and those responding only to a single inhibitor (see Supplementary material online, *Figure S9A*). When compared to cells sensitive to Ba^2+^ only, Ba^2+^ application on dual responding glomus cells resulted in no changes in Ca^2+^ event profile (see Supplementary material online, *Figure S9B*–*F*). Administering TEA + 4AP in dual responding cells shows a significant shortening of rise time when compared to cells exhibiting only TEA + 4AP-sensitivity, with no notable differences in fluorescence intensity, fall time, duration or total Ca^2+^ influx (see Supplementary material online, *Figure S9G*–*K*). Collectively, these results reveal the existence of three populations of glomus cells differentiated by their K^+^ channel expression. Furthermore, we present evidence of functional interplay between these K^+^ channels to modulate net Ca^2+^ influx, which, for the most part, shares a striking resemblance to Ca^2+^ events evoked by KCN.

### 3.5 Ba^2+^ and TEA + 4AP partially inhibit KCN-sensitivity and underly distinct components of the KCN-mediated Ca^2+^ event in glomus cells

To obtain further insight into the relationship between K^+^ channel inactivation and KCN-mediated Ca^2+^ events, we characterized Ca^2+^ events elicited by KCN in the presence of either Ba^2+^ or TEA + 4AP. First, we examined whether inactivation of TASK channels can prevent KCN-mediated Ca^2+^ events. To address this, we began by identifying which glomus cells were Ba^2+^-sensitive. Following washout, we treated the same cells with KCN but in the presence of Ba^2+^. Assuming that 100% of Ba^2+^-sensitive cells are KCN-sensitive (see Supplementary material online, *Figure S10A*), we observed a significant reduction (64%), but not complete abolition of KCN-sensitive cells in this population when blocked by Ba^2+^ (see Supplementary material online, *Figure S10C*). This suggests that inactivation of Ba^2+^-sensitive TASK channels constitutes a core component of the KCN response. Given this, we postulated that Ca^2+^ events evoked by KCN from Ba^2+^-sensitive cells would be markedly different to those from cells lacking Ba^2+^ sensitivity. Indeed, KCN-mediated Ca^2+^ events in Ba^2+^-sensitive cells exhibited significantly smaller fluorescence intensity than Ca^2+^ events evoked by KCN in glomus cells lacking Ba^2+^ sensitivity (see Supplementary material online, *Figure S10D*, *E*). While there was no difference in rise times between the two populations, Ba^2+^-sensitive cells tended to demonstrate faster fall times and shorter durations, which ultimately resulted in significantly reduced total Ca^2+^ influx (see Supplementary material online, *Figure S10F*–*I*).

Adopting a similar experimental approach as above, administering TEA + 4AP in conjunction with KCN to TEA + 4AP-sensitive glomus cells (which were also assumed to be 100% KCN-sensitive, Supplementary material online, *Figure S11A*) abolished the KCN response in 63% of cells (see Supplementary material online, *Figure S11C*). Ca^2+^ event characteristics were also different between cells that were TEA + 4AP-sensitive and insensitive. However, unlike cells exhibiting Ba^2+^ sensitivity, TEA + 4AP-sensitive cells exhibited significantly larger fluorescence intensity evoked by KCN when compared to TEA + 4AP-insensitive cells (see Supplementary material online, *Figure S11D*, *E*). This was accompanied by small changes in rise and fall times and an overall increase in duration, which caused a significant increase in net Ca^2+^ influx (see Supplementary material online, *Figure S11F*–*I*). Taken together, these findings reveal distinct and contrasting roles for Ba^2+^- and TEA + 4AP-sensitive K^+^ channels in modulating Ca^2+^ signalling in glomus cells activated by KCN.

### 3.6 Ba^2+^ drives greater tachypnea and bradycardia compared to TEA + 4AP in the *in situ* Wistar rat preparation

**Figure 5:**
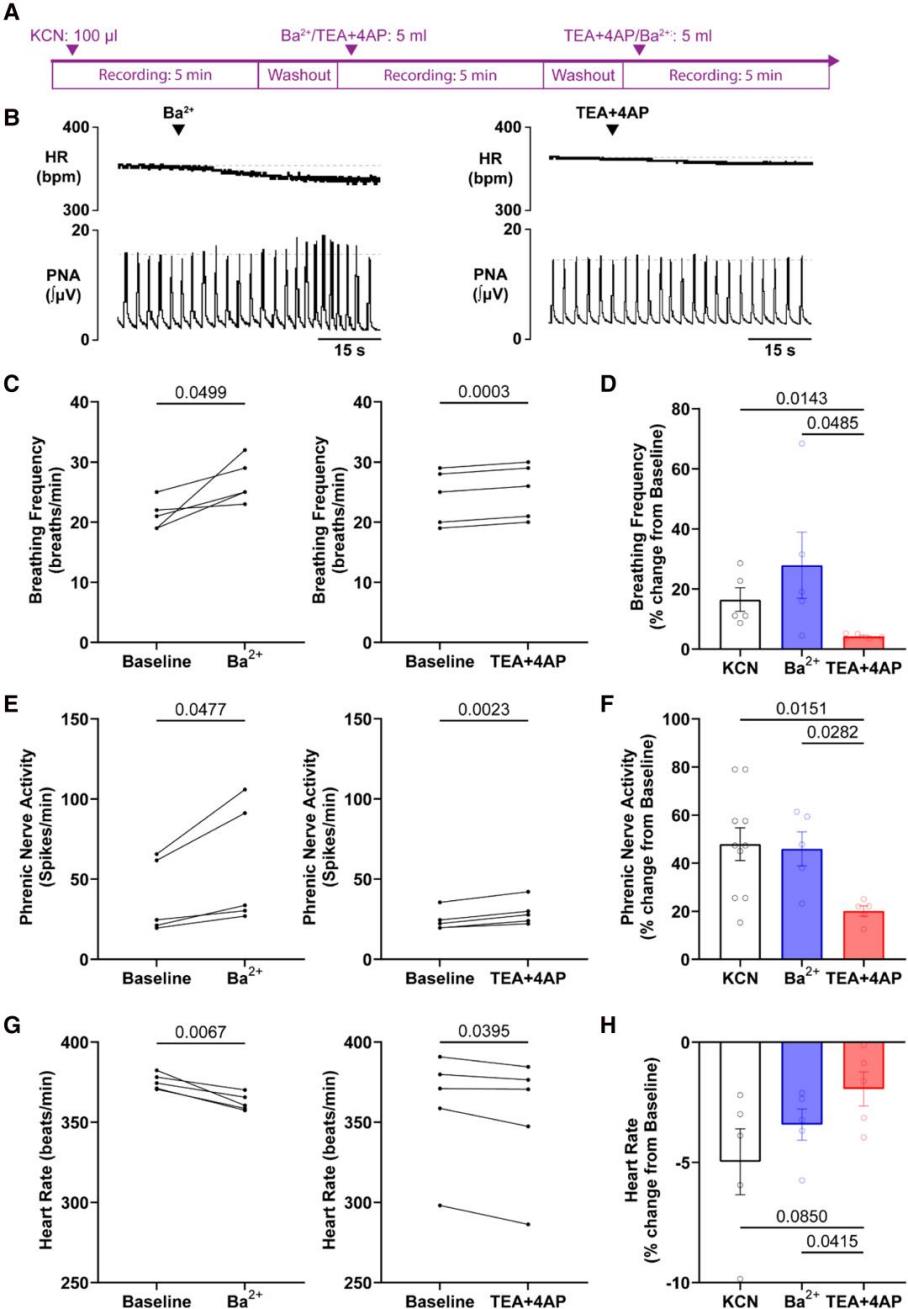
Perfusing Wistar rat CB *in situ* with Ba^2+^ or TEA + 4AP evokes distinct chemoreflex responses. (*A*) Experimental protocol used to examine the effects of KCN, Ba^2+^ or TEA + 4AP on chemoreflex responses in the dpWHBP. Washout: 10 min. (*B*) Representative traces showing changes in heart rate (HR) and phrenic nerve activity (PNA) after perfusing the CB with either Ba^2+^ or TEA + 4AP. (*C*) Increase in breathing frequency above baseline after Ba^2+^ or TEA + 4AP infusion. (*D*) Relative increase in breathing frequency from baseline induced by KCN, Ba^2+^, or TEA + 4AP. (*E*) Increase in phrenic nerve activity (firing frequency) above baseline after Ba^2+^ or TEA + 4AP infusion. (*F*) Relative increase in phrenic nerve activity from baseline induced by KCN, Ba^2+^, or TEA + 4AP. (*G*) Decrease in heart rate below baseline after Ba^2+^ or TEA + 4AP infusion. (*H*) Relative decrease in heart rate from baseline induced by KCN, Ba^2+^, or TEA + 4AP. *n* = 10 animals. Error bars are ± SEMs. *P* values were calculated using paired *t*-test.

Next, we investigated whether the cellular-level differences observed with either Ba^2+^ or TEA + 4AP translated into distinct chemoreflex responses at the systemic level. To test this, we used a double-perfused *in situ* working heart–brainstem preparation (dpWHBP), which isolates the CB vascularly, allowing for localized delivery of either Ba^2+^ (10 mM, 5 mL bolus) or TEA + 4AP (10 and 5 mM, respectively; 5 mL bolus) to the CB (*Figure 5A*) without transit of drugs into the brain.

First, administration of either blocker increased respiratory rate (*Figure 5B, C*); however, this effect was significantly greater with Ba^2+^ than with TEA + 4AP (*Figure 5D*). This tachypnoeic response was accompanied by a concomitant increase in phrenic nerve activity (firing frequency) above baseline for both treatments (*Figure 5B, E*), with the relative increase in phrenic nerve activity significantly larger following Ba^2+^ than TEA + 4AP (*Figure 5F*). Second, both interventions reduced heart rate (*Figure 5B, E*), but the bradycardic response was markedly more pronounced with Ba^2+^ (*Figure 5F*). Third, although thoracic chain sympathetic nerve activity increased with both treatments (see Supplementary material online, *Figure S12C*), this effect reached statistical significance only with Ba^2+^ (see Supplementary material online, *Figure S12A*, *B*). Fourth, both Ba^2+^ and TEA + 4AP tended to increase phrenic nerve burst amplitude (see Supplementary material online, *Figure S12D*), with no significant differences between treatments (see Supplementary material online, *Figure S12E*).

Finally, we compared Ba^2+^- and TEA + 4AP-evoked chemoreflex responses with those induced by KCN. No significant differences were observed between KCN and Ba^2+^ across any of the four measured parameters (*Figure 5D–H*; Supplementary material online, *Figure S12C*, *E*). In contrast, TEA + 4AP elicited significantly smaller increases in breathing frequency, phrenic nerve activity, and phrenic nerve amplitude compared with KCN (*Figure 5D, F*; Supplementary material online, *Figure S12E*). Similarly, the reduction in heart rate was significantly attenuated with TEA + 4AP relative to KCN.

Evidently, our data illustrate that Ba^2+^ perfusion of the CB elicits a greater tachypnoeic and bradycardic response than TEA + 4AP *in situ* and is potentially indicative of the different subpopulations of glomus cells exhibiting distinct K^+^ channels. In conjunction with *in vitro* data, these findings could support a model in which Ba^2+^-sensitive pathways are the dominant drivers of CB-evoked chemoreflex, whereas TEA + 4AP-sensitive pathways play a more limited, modulatory role in the Wistar rat.

### 3.7 Ba^2+^ and TEA + 4AP differentially alter Ca^2+^ events *in vitro* in SHRs compared to Wistar rats

To strengthen the notion that *in vitro* KCN-mediated responses observed in Wistar rats and SHRs arise from differential changes in distinct K^+^ channel expression and activity, we examined Ca^2+^ events in glomus cells from SHRs and compared these with those of Wistar rats.

**Figure 6:**
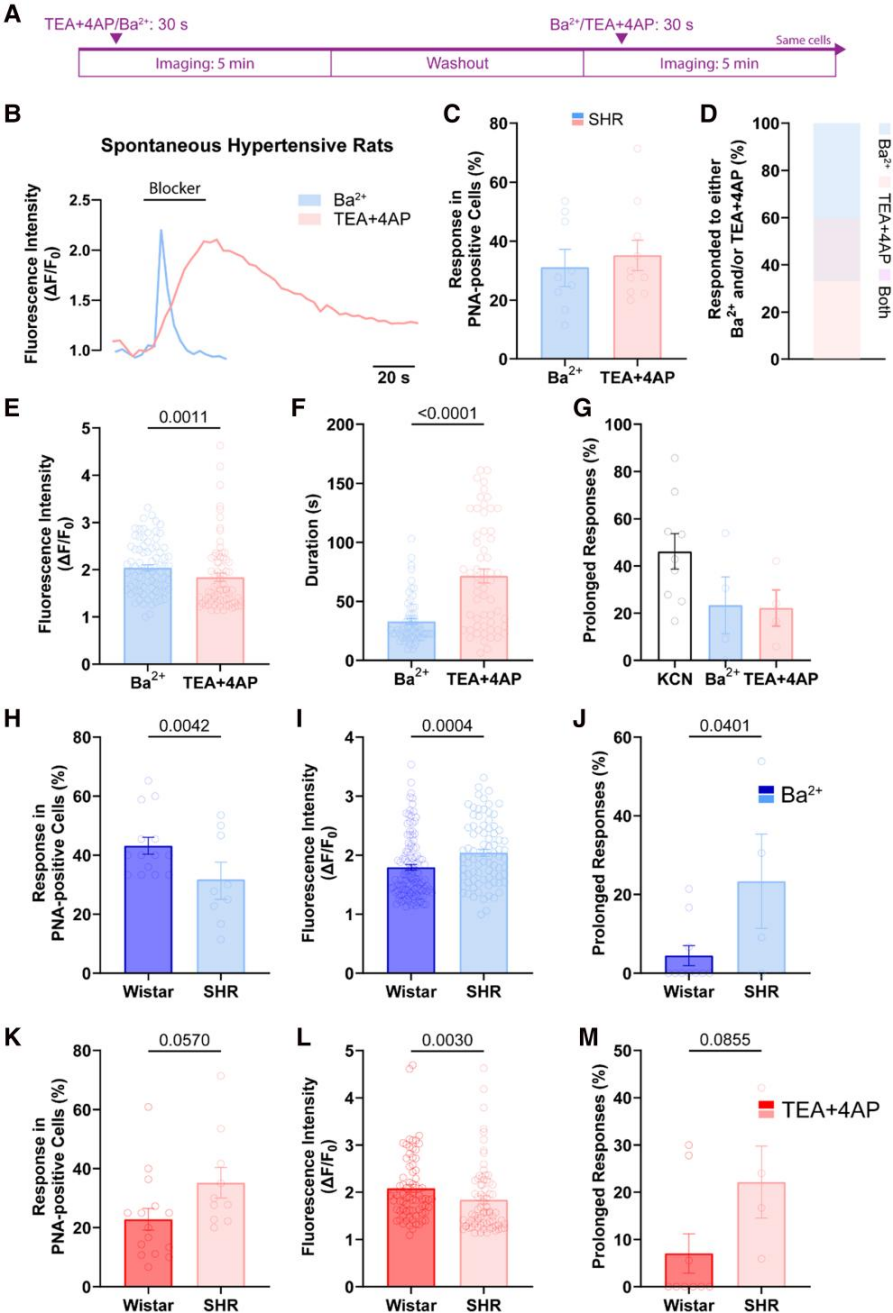
Ba^2+^ and TEA + 4AP evoke distinct Ca^2+^ events in SHRs that are differentially altered relative to Wistar rats. (*A*) Experimental confocal imaging protocol showing Ba^2+^ (10 mM) or TEA (10 mM) + 4AP (5 mM) were given in a randomized order in each run. The runs were carried out on the same set of glomus cells. (*B*) Representative traces showing two distinct types of Ca^2+^ events evoked by either Ba^2+^ or TEA + 4AP in SHR glomus cells. (*C*) Ba^2+^- or TEA + 4AP-sensitive glomus cells as a percentage of all PNA-positive cells. (*D*) Percentage of glomus cells that responded to Ba^2+^ and/or TEA + 4AP. (*E*, *F*) Ba^2+^- and TEA + 4AP-mediated Ca^2+^ event intensity and duration. (*G*) Percentage of prolonged Ca^2+^ events evoked by KCN, Ba^2+^ or TEA + 4AP. (*H*) Proportion of Ba^2+^-sensitive cells expressed as a percentage of total PNA-positive glomus cells in Wistar and SHR rats. (*I*) Fluorescence intensity of Ba^2+^-evoked transient Ca^2+^ events in glomus cells from Wistar and SHR rats. (*J*) Percentage of Ba^2+^-evoked prolonged Ca^2+^ events in glomus cells from Wistar and SHR rats. (*K*) Proportion of TEA + 4AP-sensitive cells expressed as a percentage of total PNA-positive glomus cells in Wistar and SHR rats. (*L*) Fluorescence intensity of TEA + 4AP-evoked transient Ca^2+^ events in glomus cells from Wistar and SHR rats. (*M*) Percentage of TEA + 4AP-evoked prolonged Ca^2+^ events in glomus cells from Wistar and SHR rats. For Wistar, *n* = 231 cells/5 animals; for SHR, *n* = 125 cells/4 animals. Error bars are ± SEMs. *P* values were calculated using the Mann–Whitney *U* test.

In the SHR, application of Ba^2+^ and TEA + 4AP also evoked distinct Ca^2+^ event profiles (*Figure 6B*). The proportion of Ba^2+^- and TEA + 4AP-sensitive glomus cells was not significantly different (*Figure 6C*), with the three response subpopulations: cells sensitive exclusively to Ba^2+^, exclusively to TEA + 4AP, or to both, being evenly distributed (*Figure 6D*). TEA + 4AP-sensitive cells displayed significantly lower Ca^2+^ fluorescence intensity compared with Ba^2+^-sensitive cells (*Figure 6E*). In contrast, Ca^2+^ event rise time, fall time, duration, and AUC (Ca^2+^ influx magnitude) were all markedly greater in TEA + 4AP-sensitive cells relative to Ba^2+^-sensitive counterparts (*Figure 6F*; Supplementary material online, *Figure S13A*–*C*). The incidence of prolonged Ca^2+^ responses following Ba^2+^ or TEA + 4AP application did not differ significantly from KCN stimulation and was comparable between the two blocking paradigms (*Figure 6G*).

When we compared the Ca^2+^ event profiles between Wistar rats and SHRs, we found that SHRs exhibited a shift in K^+^ channel expression and activity relative to Wistar rats, characterized by a reduced proportion of Ba^2+^-sensitive cells and an increased proportion of TEA + 4AP-sensitive cells (*Figure 6H, K*). Ca^2+^ event intensity was significantly greater in Ba^2+^-sensitive cells but significantly lower in TEA + 4AP-sensitive cells in SHRs compared with Wistars (*Figure 6I, L*). For both Ba^2+^- and TEA + 4AP-sensitive populations, the occurrence of prolonged Ca^2+^ responses was markedly augmented in the SHR (*Figure 6J, M*). Ca^2+^ event rise time was prolonged in both blocker-sensitive groups in SHRs vs. Wistars (see Supplementary material online, *Figure S13D*, *H*), whereas event duration was reduced in Ba^2+^-sensitive, but not TEA + 4AP-sensitive cells (see Supplementary material online, *Figure S13F*, *J*). Lastly, event fall time and magnitude were largely comparable between strains (see Supplementary material online, *Figure S13E*, *G*, *I*, *K*). Together, these data demonstrate a shift in K^+^ channel distribution, accompanied by remodelling of Ca^2+^ event dynamics in CB glomus cells during the early stages of hypertension development.

### 3.8 Ba^2+^ and TEA + 4AP differentially alter chemoreflex response *in situ* in SHRs compared to Wistar rats

To determine whether cellular changes in K^+^ channel expression and activity translate into strain-dependent chemoreflex responses, we characterized in situ chemoreflexes evoked by Ba^2+^ and TEA + 4AP in the dpWHBP of SHRs and compared these with responses in Wistar rats.

**Figure 7:**
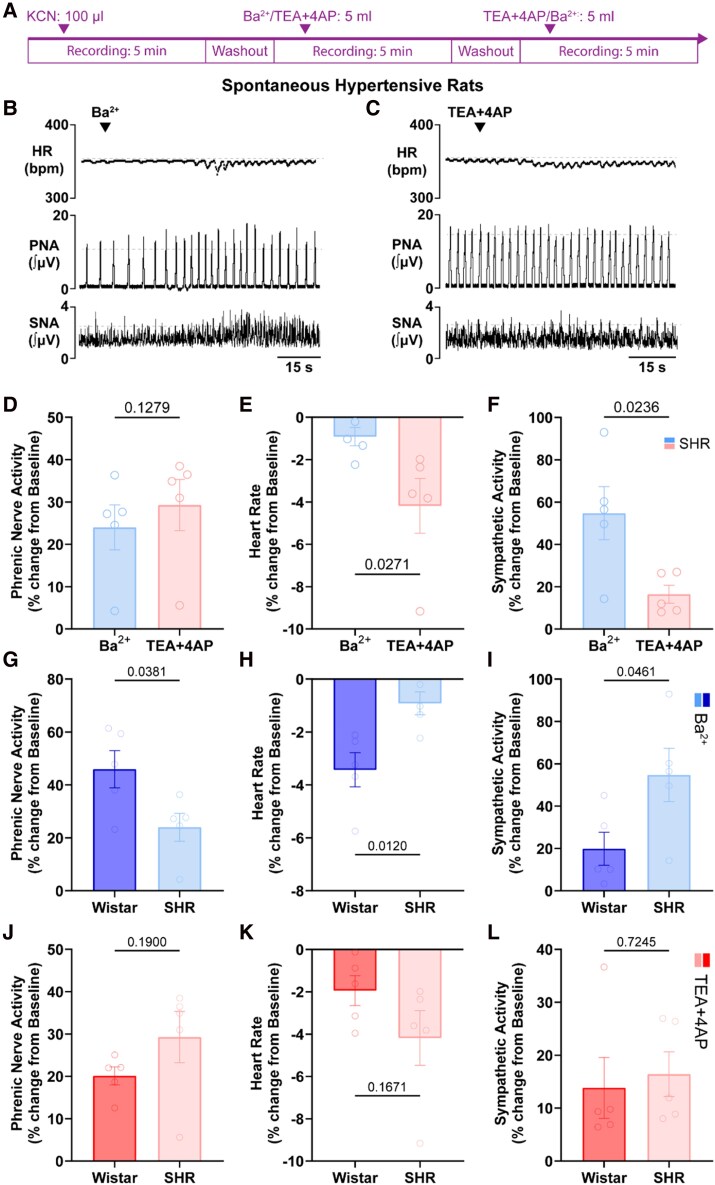
Ba^2+^- and TEA + 4AP-evoked chemoreflex responses are distinct in the SHR and are differentially altered compared with Wistar rats. (*A*) Experimental protocol used to examine the effects of Ba^2+^ or TEA + 4AP on chemoreflex responses in the dpWHBP. Washout: 10 min. (*B*) Representative traces showing changes in heart rate (HR), phrenic nerve activity (PNA), and thoracic sympathetic nerve activity (SNA) after perfusing the CB with either Ba^2+^ or TEA + 4AP. (*D*–*F*) Relative changes from baseline in phrenic nerve activity, heart rate, and sympathetic activity induced by Ba^2+^ or TEA + 4AP in SHRs. (*G*–*I*) Relative changes from baseline in phrenic nerve activity, heart rate, and thoracic sympathetic nerve activity induced by Ba^2+^ between Wistar rats and SHRs. (*J*–*L*) Relative changes from baseline in phrenic nerve activity, heart rate, and thoracic sympathetic nerve activity induced by TEA + 4AP between Wistar rats and SHRs. For Wistar, *n* = 5 animals; for SHR, *n* = 5 animals. Error bars are ± SEMs. *P* values were calculated using unpaired *t*-test.

In SHRs, Ba^2+^ and TEA + 4AP elicited distinct *in situ* chemoreflex profiles (*Figure 7B*). Ba^2+^ administration produced significantly greater increases in breathing frequency, phrenic nerve amplitude, and thoracic sympathetic nerve activity compared with TEA + 4AP (*Figure 7F*; Supplementary material online, *Figure S14A*, *B*). In addition, the reduction in heart rate was significantly smaller following Ba^2+^ treatment (*Figure 7E*). No significant differences were observed between Ba^2+^ and TEA + 4AP in phrenic nerve activity (*Figure 7D*).

Comparison with Wistar rats revealed marked strain-dependent differences. In SHRs, Ba^2+^-mediated increases in phrenic nerve activity and reductions in heart rate were significantly blunted relative to Wistars (*Figure 7G, H*). Conversely, TEA + 4AP-mediated increases in phrenic nerve activity and decreases in heart rate tended to be augmented in SHRs (*Figure 7J, K*). Thoracic sympathetic nerve activity was significantly enhanced by Ba^2+^, but not by TEA + 4AP, in SHRs compared with Wistar rats (*Figure 7I, L*). Neither Ba^2+^ nor TEA + 4AP significantly altered breathing frequency or phrenic nerve amplitude between strains (see Supplementary material online, *Figure S14C*–*F*). Together, these data highlight strain-dependent differences in Ba^2+^- and TEA + 4AP-sensitive K^+^ channel and their contribution to CB-driven chemoreflex outputs.

### 3.9 Wistar and SHR CB differentially express K^+^ channel genes

Finally, we examined whether the functional differences observed at the cellular and tissue levels between Wistars and SHRs were accompanied by alterations in the expression of TASK and BK/Kv channel genes. To this end, we performed comparative analyses using our previously published bulk RNA-sequencing transcriptomic dataset from rat CBs (GEO accession: GSE178504).^38^ Expression levels of TASK and BK/Kv channel genes were compared between the two strains.

These included *KCNK3* (TASK-1), *KCNK9* (TASK-3), *KCNMA1* (BK), *KCNH2* (voltage-gated hERG), *KCNQ5* (Kv7.5), and *KCNC3* (Kv3.3).^39–43^ Relative to Wistar rats, expression of both *KCNK3* and *KCNK9* was reduced in SHR, with the decrease reaching statistical significance for KCNK9. KCNMA1 expression was comparable between strains. In contrast, expression of the Kv channel-encoding genes *KCNH2*, *KCNQ5*, and *KCNC3* was significantly increased in SHR compared with Wistar rats. Together, these transcriptional differences are consistent with the functional alterations observed and support the presence of altered K^+^ channel sensitivity in the hypertensive CB.

### 3.10 Sex differences in KCN-mediated glomus cell Ca^2+^ signalling

To extend the translational relevance of our findings, we examined KCN-mediated Ca^2+^ events in glomus cells from young female Wistar rats and compared these responses with those of age-matched males. No significant sex differences were observed in overall fluorescence intensity or rise time when transient and prolonged Ca^2+^ events were considered collectively (see Supplementary material online, *Figure S16B*, *C*). Similarly, the relative proportion of transient vs. prolonged Ca^2+^ events was comparable between female and male Wistars (see Supplementary material online, *Figure S16D*).

When analysed by event subtype, however, clear sex-dependent differences emerged. Transient Ca^2+^ events in females displayed significantly greater fluorescence intensity and magnitude (AUC), accompanied by lengthened event duration and fall time compared with males (see Supplementary material online, *Figure S16E*–*J*). In contrast, prolonged events did not differ in peak intensity between sexes, but exhibited a significantly faster rise time in females (see Supplementary material online, *Figure S16K*–*M*). These findings indicate a subtle, but distinct sex-dependent modulation of CB Ca^2+^ signalling in response to KCN stimulation.

## 4. Discussion

Given the multimodal nature of the CB, we proposed the existence of distinct pathways for eliciting different components of the peripheral chemoreflex in response to distinct external stimuli.^8^ This is consistent with a recently proposed ‘ribbon cable’ hypothesis of connectivity between different sub-groups of glomus cells and distinct chemoreflex pathways. What these pathways are and how they are activated remain poorly understood in both health and disease. In this study, we describe for the first time distinct populations of glomus cells defined by their unique K^+^ channel expression (Ba^2+^-sensitive TASK channels and TEA + 4AP-sensitive BK/Kv channels) that preferentially mediate chemoreflex responses. Importantly, in the prehypertensive CB, these channels undergo divergent changes in expression and activity, providing a cellular basis for heightened sympathetic drive.

We have found that application of KCN, a hypoxia mimetic, to the CB *ex vivo* generates a biphasic response from the CSN. When compared to the Wistar, we report an overall augmented response magnitude in the SHR, with the two response components being disproportionately affected in disease. We propose that each component of this response may be attributed to distinct populations of chemosensory glomus cells exhibiting either transient or prolonged KCN-mediated Ca^2+^ events. Importantly, we found glomus cells with KCN evoked transient and prolonged [Ca^2+^]_i_ responses analogous to CSN responses. We also show how the overall proportion of these cell types is shifted towards having more prolonged Ca^2+^ events in the SHR, which may contribute to CB hyperexcitability.^7^ Finally, we provide compelling *in vitro* and *in situ* evidence that the inactivation and interaction of different K^+^ channels (TASK or BK/Kv) drive different chemoreflex response patterns.

In previous studies where we had utilized the *ex vivo* CB-CSN preparation, we noted a biphasic CSN response to KCN application.^5,31^ Here, we demonstrated that this response could be robustly reproduced in both Wistar and SHR and is comprised of two components with distinct dynamic and kinetic profiles. The combined magnitude of the integrated response was augmented in SHRs compared to Wistars, consistent with what has been reported in the literature when animals were subjected to either cyanide or hypoxia.^7,44–46^ However, this augmentation observed in the SHR is disproportionate as it was significantly greater only in the 2nd component of the biphasic CSN response (*Figure 1D*). We speculate that under physiological conditions, the 1st component serves as the primary response to KCN, while the 2nd component modulates this activity. Such mechanism could enable fine-tuning of CSN afferent discharge, allowing for a more adaptive firing pattern in response to different stimuli. The relative difference in magnitude between the 1st and 2nd components of the CSN response is lost in disease, potentially contributing to the aberrant sympathetic activity seen in hypertension.^3,11^ Comparable mechanisms are evident in synaptic transmission involving receptors with opposing actions, such as GABA_A_/GABA_B_. For example, upon GABA exposure, rapid activation of GABA_A_ receptors transiently enhances synaptic transmission, which is subsequently suppressed by the slower-activating GABA_B_ receptors, producing a brief window of enhanced activity.^47^ Fine-tuning of this excitation-inhibition balance depends on the relative expression of presynaptic GABA_A_/GABA_B_ receptors, which can become disrupted under pathophysiological conditions such as epilepsy, where reduced GABA_B_ receptor expression has been reported.^48^

Changes in receptor expression on CSN afferents could shed light on these findings. For example, Pijacka *et al.* revealed significant upregulation of the excitatory purinergic P2X3 receptor on afferent neurons from the SHR.^7^ It would be equally interesting to determine whether the above changes could also result from a shift in afferent fibre structure and/or function. Morphological studies of the rat CSN reveal it is predominantly composed of unmyelinated C-fibres (86.3%),^49^ and that these fibres respond differently when stimulated compared to their myelinated counterpart.^50^ Whether these fibre types preferentially connect to distinct glomus cell subpopulations remains an important question for further investigation.

### 4.1 Altered K^+^ channel-dependent Ca^2+^ signalling in Wistar and SHR

When glomus cells become stimulated in conditions such as hypoxia, they depolarize. This results in an increase in intracellular Ca^2+^, which subsequently drives the release of neurotransmitters onto the petrosal afferent terminals.^7,13–18^ Here, we discovered that ∼74% of all glomus cells from Wistar CBs exhibited a transient Ca^2+^ event to KCN, while the remainder displayed a markedly prolonged response, which was significantly greater in fluorescence intensity. Paradoxically, the intensity of both transient and prolonged Ca^2+^ events was attenuated in the SHR, but due to a significant increase in the proportion of cells expressing prolonged events, the combined total Ca^2+^ influx is predicted to be potentiated. Notably, this shift coincides with the shift in integrated CSN response from being 1st component-dominant in the Wistar, to being even in the SHR (*Figure 1H*). A more detailed examination of whether distinct glomus cell subpopulations preferentially release specific neurotransmitters may help elucidate the mechanisms underlying the KCN-mediated biphasic CSN response in both physiological and pathological states.

It is widely accepted that hypoxia inhibits K^+^ conductance to evoke glomus cell depolarization,^25,51,52^ thus differences in cellular expression of K^+^ channels inevitably influence Ca^2+^ influx. In this study, we used either Ba^2+^ or TEA + 4AP to inhibit TASK or BK/Kv channels, respectively.^32–34^ Despite conflicting reports,^52–54^ the addition of either inhibitor in our hands consistently evoked Ca^2+^ events in glomus cells with distinct response profiles which, for the most part, mirror the KCN-mediated Ca^2+^ events—both in their kinetics and relative distribution across the glomus cell population. Furthermore, we demonstrate contrasting differences in KCN-mediated Ca^2+^ events between cells sensitive and insensitive to either Ba^2+^- or TEA + 4AP, signifying an interplay between different K^+^ channels and downstream Ca^2+^ influx, and subsequent neurotransmitter release. For example, Trapp *et al.* previously reported reduced hypoxia-induced CSN discharge in TASK-1-deficient mice.^55^ This finding is largely in agreement with our observation that Ba^2+^-sensitive cells exhibit significantly reduced KCN-mediated Ca^2+^ influx compared with Ba^2+^-insensitive cells (*Figure 5G*).

Extending these observations, we demonstrate that Ba^2+^- and TEA + 4AP-sensitive pathways are differentially engaged in the hypertensive CB. In SHR, K^+^ channel blockade revealed distinct Ca^2+^ event profiles within glomus cells, and comparison with Wistar rats uncovered a shift in K^+^ channel expression and activity. Specifically, Ba^2+^-sensitive cells exhibited augmented Ca^2+^ event intensity but with reduced expression, whereas TEA + 4AP-sensitive cells showed diminished Ca^2+^ event intensity but increased expression. Work by Tan *et al.* reported increased acid-evoked TASK-1 current from SHR vs. Wistar glomus cells, consistent with our functional observations.^56^ However, their reported overexpression of TASK-1 and TASK-3 contrasts with our RNA-seq data, which indicate reduced TASK and increased Kv channel expression in SHRs. Notably, both studies assessed gene expression at the level of the whole CB, which may not reflect cell-specific changes. Collectively, these data suggest that TASK and BK/Kv channel inhibition activates distinct intracellular signalling pathways, and that the onset of hypertension is accompanied by a functional reweighting of TASK vs. BK/Kv channel influence on glomus cell excitability, driving the attenuated KCN-mediated Ca^2+^ event intensity in SHR glomus cells.

Our results reinforce that the central role of K^+^ conductance is crucial in driving depolarization in response to KCN, as blockade of these channels significantly reduced the proportion of glomus cells sensitive to KCN in Wistar rats. However, this reduction was only partial, likely reflecting the contribution of other hypoxia-sensitive channels such as TRP^57,58^ and TREK^59^ and parallel signalling pathways. Importantly, K^+^ channel inhibition alone cannot account for the prolonged Ca^2+^ events evoked by KCN. We also saw that the incidence of prolonged Ca^2+^ events in both Ba^2+^- and TEA + 4AP-sensitive cells was markedly higher in SHRs compared to Wistars. Given that cyanide inhibits cytochrome c oxidase and disrupts mitochondrial oxidative phosphorylation, mitochondrial mechanisms may contribute to these prolonged responses.^60^ Indeed, substantial heterogeneity in mitochondrial morphology and function has been reported both between and within individual cells.^61–63^ Moreover, altered mitochondrial activity has been described in the brain,^64^ liver,^65^ and kidney^66^ of young SHRs relative to normotensive controls. It is therefore plausible that subpopulations of glomus cells exhibit mitochondrial differences that facilitate distinct Ca^2+^ signalling in health and early stages of hypertension.

### 4.2 Altered K^+^ channel-dependent chemoreflex response in Wistar and SHR

A key finding from this study is that distinct K^+^ channels drive different chemoreflex responses at the systemic level, and that this organization is altered at the onset of hypertension. This supports the ribbon cable hypothesis that glomus cells are connected to distinct afferent pathways that can mediate separate components of the chemoreflex response.^8^ Our findings also have important implications for understanding the oxygen-sensing role of the CB and its broader multimodal chemosensory function.

In normotensive conditions, oxygen detection in the CB is traditionally attributed to the inhibition of background K^+^ channels, such as TASK, during hypoxia, leading to glomus cell depolarization, Ca^2+^ influx, and neurotransmitter release.^32^ Our observation that TASK channel blockade with Ba^2+^ evokes more pronounced tachypnoeic and bradycardic responses than inhibiting BK/Kv channels suggests that TASK channels play a dominant role in the respiratory response to hypoxia. The larger proportion of Ba^2+^-sensitive glomus cells (outnumbering TEA + 4AP-sensitive glomus cells by roughly two to one, *Figure 4E*) supports the idea that TASK-mediated signalling forms a primary pathway for maintaining blood oxygenation.^32^ At the same time, the distinct pattern of responses to TEA + 4AP indicates that other K^+^ channel subtypes, while less prevalent, contribute to pathways supporting the CB’s ability to differentially modulate effectors in response to different physiological stimuli.^8,34^ The fact that Ba^2+^, but not TEA + 4AP, significantly increased thoracic sympathetic nerve activity further points to functional specialization, including connectivity among glomus cell subpopulations.

Extending these observations to hypertension, we show that TASK- and BK/Kv-sensitive pathways retain distinct functional signatures, but are differentially engaged in the SHR. Comparison with Wistar rats revealed a marked strain-dependent divergence: Ba^2+^-mediated increases in phrenic nerve activity and bradycardia were significantly blunted in SHRs, whereas TEA + 4AP-mediated effects on these same outputs were exaggerated. In addition, sympathetic nerve activity was selectively augmented by Ba^2+^, but not TEA + 4AP, in SHRs. These changes likely reflect altered activity and redistribution of TASK and BK/Kv channels within the hypertensive CB, and that TASK-sensitive pathways remain a dominant driver of above chemoreflex outputs, as observed in Wistar rats. Consistent with this interpretation, Grundy *et al*. showed depressed phrenic nerve activity when mean arterial pressure was increased in artificially ventilated dogs.^67^ One clinical study showed that individuals with persistently elevated heart rate in early-stage hypertension have a markedly increased risk of developing sustained hypertension.^68^ The aberrant increase in sympathetic nerve activity in hypertension is also well-established.^3,4^

### 4.3 Sex-specific differences in KCN-mediated glomus cell Ca^2+^ signalling

For the first time, we demonstrate that KCN evokes distinct Ca^2+^ events in glomus cells from male and female Wistar rats, with events in females generally being faster, larger in magnitude, and longer in duration than those observed in males. These differences may reflect modulation by sex hormones, particularly oestrogen and progesterone, which are known to influence K^+^ channel function^69–71^ and mitochondrial signalling.^72,73^

Although no exogenous hormones were applied in our experimental setup, Joseph *et al.* identified the mitochondrial P450 side-chain cleavage enzyme, a mitochondrial enzyme crucial for sex hormone synthesis, within the CB.^74^ This finding supports the hypothesis that the CB may locally synthesize progesterone, which could act as an autocrine modulator of peripheral chemoreceptor function and glomus cell excitability.

The precise relationship between the *in vitro* sex-specific differences and downstream chemoreflex responses remains to be fully elucidated. However, several studies have shown that progesterone and estrogen enhance catecholaminergic signalling within the CB, thereby augmenting chemoafferent drive and improving ventilatory adaptation to hypoxia.^75–77^ Thus, these mechanisms may provide a molecular basis for the higher prevalence of sleep apnoea in adult men compared with women,^78^ and the increased risk observed in women following menopause.^79^

### 4.4 Limitations of the study

We recognize that while the use of KCN does not fully mimic hypoxia-induced response in the CB and glomus cells, it is conceptually important as it targets several common hypoxia-induced pathways in glomus cells and the CB.^80^ We employed KCN because it acts rapidly and can be delivered in small, controlled volumes, allowing precise, intermittent stimulation and consistent evocation of discrete Ca^2+^ events and chemoafferent responses. This temporal precision was critical for identifying the biphasic pattern we observed—features that would likely be obscured by the slower onset of hypoxia. Nonetheless, future studies using direct hypoxic stimulation would further strengthen these findings.

We chose to study juvenile SHRs to investigate early mechanisms underlying hypertension rather than secondary adaptations to chronic disease. At this age, SHRs experience the early onset of hypertension, thereby minimizing confounding effects from long-standing elevations in blood pressure, end-organ damage, and structural remodelling.^81^ While blood pressure does not reach hypertension levels in the juvenile SHRs, it can already be significantly higher than age-matched normotensive counterparts.^82^ Furthermore, before established hypertension is present, the peripheral chemoreceptor reflex is already hyperactive in juvenile SHRs.^7^ This is evidenced by an enhanced sympathetic nerve activity response to cyanide compared to age-matched controls.^56^ Thus, we believe using juvenile SHRs allows clearer attribution of the observed alterations in CB signalling and K^+^ channel sensitivity to primary, genetically driven mechanisms relevant to the initiation of hypertension.

The use of non-ratiometric indicator Fluo-4 limited our ability to directly quantify [Ca^2+^]_i_. In principle, it is possible that the smaller, prolonged Ca^2+^ events in the SHR cells could be attributed to a greater baseline Ca^2+^ concentration. However, the similarity in event morphology across pharmacological conditions and the consistency of strain-dependent differences argue against a simple ‘ceiling’ effect as the sole explanation. Nonetheless, future studies employing ratiometric Ca^2+^ imaging will be required to directly quantify [Ca^2+^]ᵢ and to define its precise relationship with K^+^ channel activity under physiological and hypertensive conditions.

While comparative analysis of K^+^ channel gene expression between strains provides insight into overall changes in K^+^ expression in the hypertensive CB, the RNA-seq data were derived from whole CBs and are therefore not restricted to glomus cells. Consequently, further investigations employing cell-specific approaches, such as immunofluorescence staining for individual TASK and BK/Kv channels, will be required to determine whether distinct glomus cell subpopulations differentially express specific K^+^ channel subtypes. Similarly, electrophysiological approaches such as patch-clamp recordings or targeted gene knockdown/silencing would enable a more direct assessment of K^+^ channel activity across distinct glomus cell populations in both health and disease.

In summary, we have expanded the notion that there are distinct subpopulations of glomus cells in the CB characterized by discrete intrinsic ion conductance, as well as unique morphological^26,27^ and functional properties.^28–30^ The study also helps reconcile conflicting reports on the different K^+^ channels implicated in oxygen sensing in the CB.^52,54^ Based on our findings, we believe that transient and prolonged Ca^2+^ events evoked by KCN, and by proxy hypoxia, could depend on the expression of different K^+^ channel types. The magnitude of the Ca^2+^ influx dictates and modulates neurotransmission between the CB and CSN under physiological conditions. Differing Ca^2+^ levels may control which transmitters are released, thereby evoking distinct chemoreflex responses downstream. However, the increase in the number of glomus cells exhibiting prolonged Ca^2+^ events in SHR (vs. Wistar rats) may ultimately contribute to the hyperexcitability of the CB and underlying sympatho-overexcitation seen in neurogenic hypertension.

Translational perspective We reveal that distinct glomus cell subpopulations, defined by their K^+^ channel expression and Ca^2+^ dynamics, differentially contribute to carotid body (CB) signalling and chemoreflex control. In hypertension, the altered balance between these populations amplifies chemosensory drive and may underlie aberrant sympathetic activation. Understanding this cellular heterogeneity provides a new framework for targeting selective pathways within the CB rather than globally suppressing its entire functionality. Such an approach may preserve essential chemoreflex functions while reducing pathological sympathetic overactivity, offering promising avenues for novel therapeutic strategies in cardiovascular and metabolic diseases.

## Supplementary Material

cvag118_Supplementary_Data

## Data Availability

The data underlying this article will be shared on reasonable request to the corresponding author.
