# Supplementary material for: Glomus cell heterogeneity underpins distinct carotid body chemoreflex pathways: implications for hypertension
Source: Cardiovasc Res. 2026 May 24;122(10):1391–407. doi: 10.1093/cvr/cvag118 (PMC13355836; doi:10.1093/cvr/cvag118)
Supplement: cvag118_Supplementary_Data [file cvag118_supplementary_data.docx]

**Supplementary Data**

**Materials and Methods**

**Carotid sinus nerve-carotid body *ex vivo* preparation and analysis**

Animals were anesthetized with 5% (v/v) isoflurane in O2 (1 L/min) and euthanized by cervical dislocation. The left and right carotid artery bifurcations, including the CSN and CB, were exposed via a longitudinal ventral neck incision, with salivary glands and sternomastoid and sternohyoid muscles retracted or removed. The bifurcations were surgically excised and placed in a recording chamber perfused with Ringer’s solution containing (in mM): NaCl 125, NaHCO_3_ 24, KCl 3.75, CaCl_2_ 2.5, MgSO_4_ 1.25, KH_2_PO_4_ 1.25, and D-glucose 10. The solution was carbogen-gassed (5% CO_2_, 95% O_2_), heated to 37°C, filtered (25 µM nylon mesh), and perfused at a rate of 5 mL/min using a peristaltic pump (Minipuls 3, Gilson). Under a dissecting microscope, the common carotid artery was cannulated with PE-10 tubing for perfusion with Ringer’s solution, after which the CSN was carefully isolated.

Nerve activity was recorded using a bipolar glass suction electrode (1B150F-4, WPI), amplified ×10,000 (AM Systems 1700), band-pass filtered (100 Hz–1 kHz), and digitized at 20 kHz (Micro1401-3, CED). Data acquisition and analysis were performed using Spike2 software (CED). CB chemosensitivity was quantified as the percentage change in area under the curve (AUC) of the integrated CSN activity in response to KCN bolus (0.08%; 100 µL) compared to AUC of baseline integrated CSN activity of equal duration immediately prior to the stimulus. The baseline period used for each calculation was the same time-length as the CSN discharge. At the end of the recording, KCN was used to assess CB viability, and lignocaine (0.2 mL bolus; Nopaine 20 mg/mL) delivered to record the baseline noise level. Supplement Figure 1 illustrates how various integrated parameters were calculated for the 1st or 2nd component in response to KCN.

**Glomus Cell Dissociation**

Dissociation of glomus cells was performed as described previously^1^. Briefly, CBs were surgically excised in chilled, O_2_-equilibrated HEPES-Tyrode (HT) solution of the following composition (in mM): 140 NaCl, 5 KCl, 1.1 MgCl_2_, 5 Glucose and 10 HEPES. Following excision, CBs were enzymatically digested in Ca^2+^/Mg^+^-free HBSS (14170120, ThermoFisher) containing 2 mg/mL collagenase type IV (17104019, ThermoFisher), 0.4 mg/mL trypsin (T4799, Sigma-Aldrich) and 0.03 mg/mL DNase II (D4527, Sigma-Aldrich) for 60 min at 37°C. At 15, 30, 45 and 60 min, CBs were mechanically agitated for 2 min by repeated aspiration through a fire-polished Pasteur pipette, followed by the addition of 50 µM bovine serum albumin (pH100, pH Scientific) to terminate digestion. Cells were subsequently pelleted at 200 rcf, 4°C for 10 min (Centrifuge 5418R, Eppendorf) and transferred to Poly-D-Lysine (A3890401, ThermoFisher) coated 13 mm round coverslips (MAR0111530, Marienfeld Superior) for 1 hour before Ca^2+^ imaging.

**Glomus Cell Ca^2+^ Imaging and Analysis**

GCs were loaded with Fluo-4 AM (5 µM for 15 min, ab241082, Abcam) and peanut agglutinin (PNA, 90 µM for 30 min, Vector Laboratories) at room temperature and imaged using an upright Olympus FV3000 confocal microscope (Olympus) using a 40x water dipping objective (LUMPLFLN40 XW, NA 0.8, Olympus). Cells were superfused at a rate of 1.5 mL/min via a mechanised syringe pump (Minipuls3, Gilson), delivering Ca^2+^-containing (2 mM) HT solution at 32°C. Each set of recording was 5 min in duration, comprising of 93 frames at 3.22 s/frame and frame size of 1024 x 1024 pixels. Each cell was subjected to 2 sequential stimuli depending on experimental protocol in a randomised order, with a washout period of 10 min in between imaging runs. Random field of views were selected for each imaging run, and a high potassium solution (50 mM, Supplement Fig. 2) was used to assess cell viability.

Ca^2+^ events were analysed using custom-written FIJI macros whereby a region of interest is drawn around each cell. Event parameters included intensity (ΔF/F_0_, change in peak fluorescence, F, above background, F_0_), rise time (time to peak fluorescence), fall time (recovery time from peak fluorescence to baseline), event frequency, duration and total Ca^2+^ influx (area under the curve) were measured.

**Cell treatments**

For all glomus cell experiments, pharmacological interventions were applied for a period of 30 seconds. Vehicle controls used to dilute the drugs (HT and DMSO, Supplement Fig. 3) were assessed using a similar protocol to ensure the absence of fluorescence response. Drug reversibility and reproducibility on glomus cells were similarly tested using protocol outlined in Supplement Figure 4A.

**Double perfused Working Heart-brainstem Preparation (dpWHBP)**

We adapted the working heart-brainstem preparation (WHBP)^2^ to enable vascular isolation of the carotid bifurcation, hereafter referred to as the double-perfused WHBP (dpWHBP). Juvenile rats (3-6 weeks, 50-80 g) were deeply anesthetized with isoflurane (5% in O_2_, 1 L/min) until loss of paw and tail withdrawal reflexes, heparinized (350 IU i.p.; Pfizer), and euthanized by exsanguination following diaphragmatic transection. After cooling the upper body in Ringer’s solution, animals were decerebrated pre-collicularly, the lungs removed, and the descending aorta cannulated with a double-lumen catheter for retrograde perfusion. Viability was confirmed by recovery of a ramp-like phrenic nerve discharge pattern characteristic of eupnoea^2^.

The perfusate was Ringer’s solution containing 1.5% polyethylene glycol (Sigma-Aldrich), gassed with carbogen (95% O_2_, 5% CO_2_), maintained at 32°C, filtered (25 µm nylon mesh, Millipore), and recirculated. Aortic pressure was monitored via the second catheter lumen (NL108T2, Digitimer) and maintained at 55-90 mmHg by adjusting pump flow (20-25 mL/min; Watson-Marlow 530s) and supplementing vasopressin (2 nM, Sigma-Aldrich). Neuromuscular blockade was achieved with vecuronium bromide (10 mg/mL, Mylan).

For selective perfusion of the right carotid bifurcation, a second pump delivered perfusate to the right common carotid artery. The common, internal and external carotid arteries were cannulated with polyethylene tubing, while the occipital and pharyngeal branches were clamped to isolate CB circulation. Perfusion pressure was maintained at 60-100 mmHg, matched to the aortic pressure, and monitored via an internal carotid cannula connected to a second pressure transducer (NL108T2, Digitimer). Outflow from the isolated bifurcation drained through an external carotid cannula and was either recirculated back to the reservoir or diverted to a waste beaker when drugs were added

Phrenic nerve and thoracic sympathetic chain (T13-L3) activities were recorded simultaneously with bipolar suction electrodes, and heart rate was derived from inter-R-wave intervals of the ECG (forelimb electrodes). Signals were amplified (×10,000; A-M Systems 1700), band-pass filtered (10 Hz-5 kHz), digitized at 20 kHz (Micro1401-3, CED), and acquired in Spike2 (CED). Background noise (measured 15 min after pump cessation) was subtracted from sympathetic signals. Chemoreflex responses were expressed as percentage changes from baseline, with baseline duration matched to each drug response.

**Statistical analysis**

Power analysis was performed using G*Power^3^ to determine sample sizes based on known variability of measured parameters. No data were excluded from the analyses. All data were analysed using GraphPad Prism (10.4.1). Data sets were tested for normality of distribution using the Shapiro-Wilk normality test. For normally distributed data, differences were analysed using paired or unpaired Student’s t-test. For non-normally distributed data, differences were analysed using either Mann-Whitney U tests for unpaired comparisons or Wilcoxon matched-pairs signed rank test for paired comparisons. The results are expressed as the mean ± SEM unless otherwise stated. Differences were considered statistically significant when *p*<0.05. Statistical tests performed for each dataset are indicated in the figure legends.

**Supplement Figures**


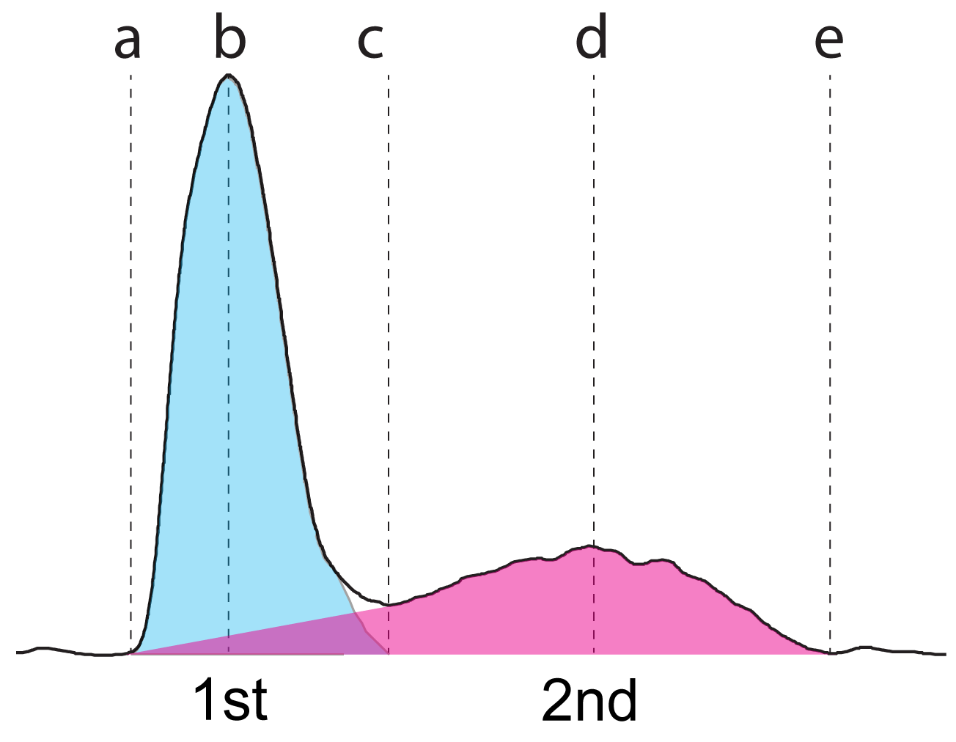


**Supplement Figure 1.** Diagram showing how different integrated parameters are calculated for the 1st or 2nd component of the CSN response evoked by KCN. Amplitude=b or d; Area under the curve (AUC)=½×(c-a)×b or ½×(e-a)×d; Rise time=a to b or a to d; Fall time=b to c or d to e; Duration=a to c or a to e; Time between peaks=d-b; Amplitude ratio: d/b; AUC ratio=1st AUC/2nd AUC.


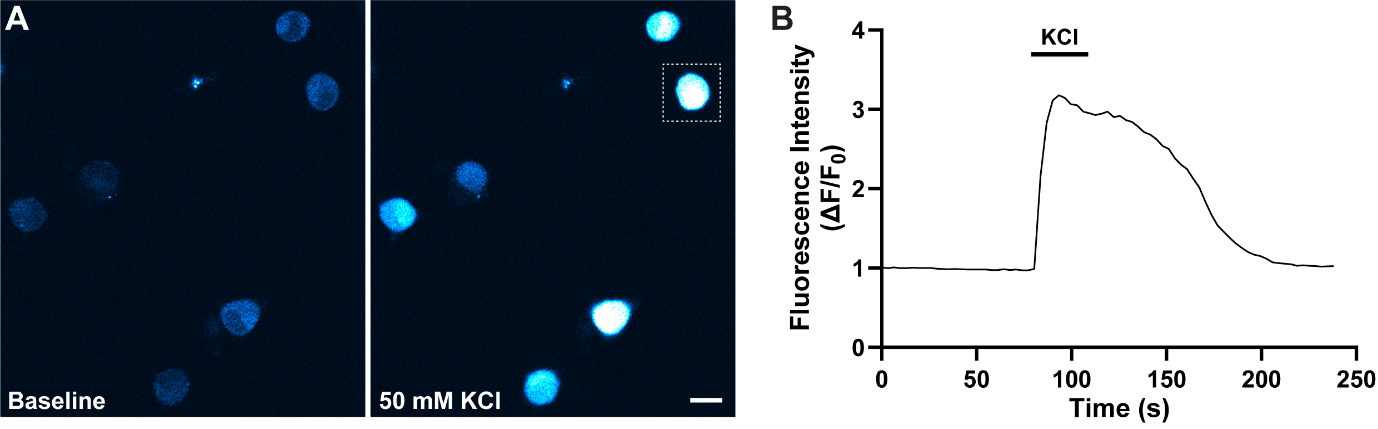


**Supplement Figure 2. Assessing dissociated glomus cells viability using high potassium buffer.** (A) Representative confocal images of Fluo-4 loaded glomus cells at baseline and after KCl (50 mM) application. (B) Trace showing changes in fluorescence intensity of a cell (outlined by dashed box in A) after KCl.

**
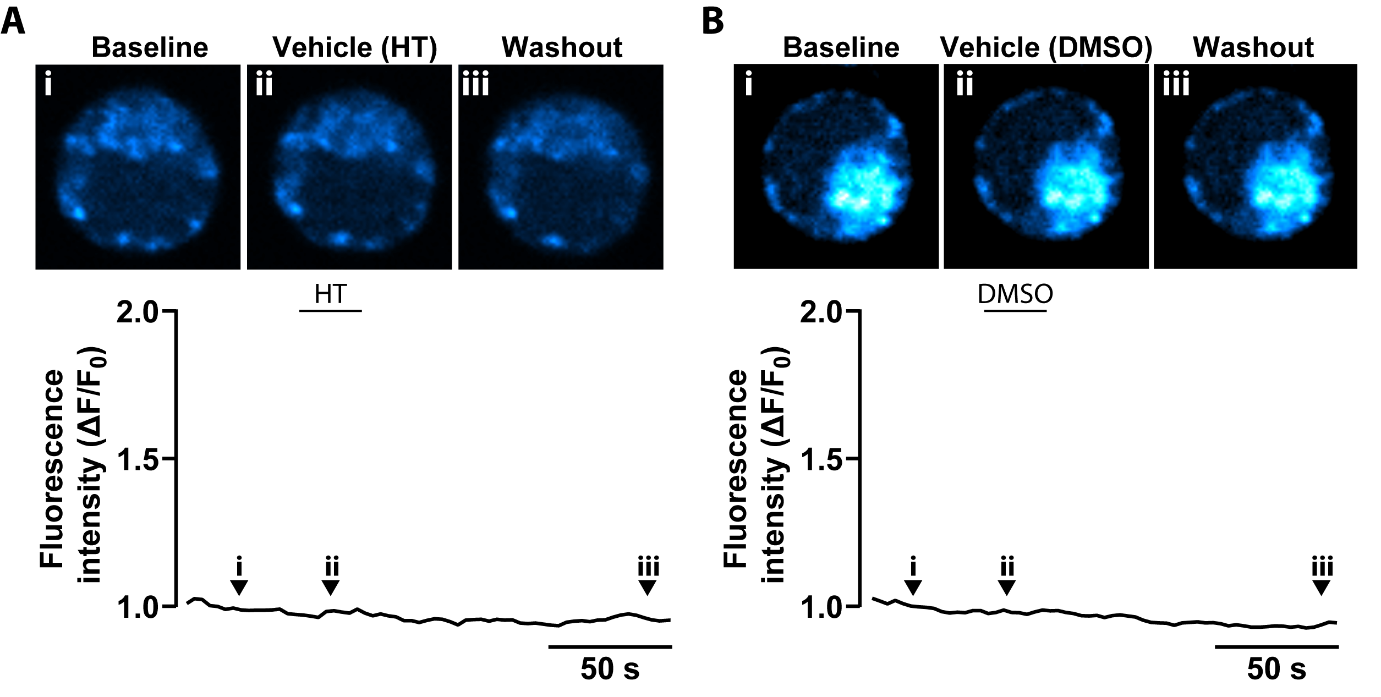
**

**Supplement Figure 3.** Vehicle controls (A) HEPES-Tyrodes and (B) DMSO do not evoke fluorescence Ca^2+^ events when superfused over Fluo-4 loaded glomus cells.


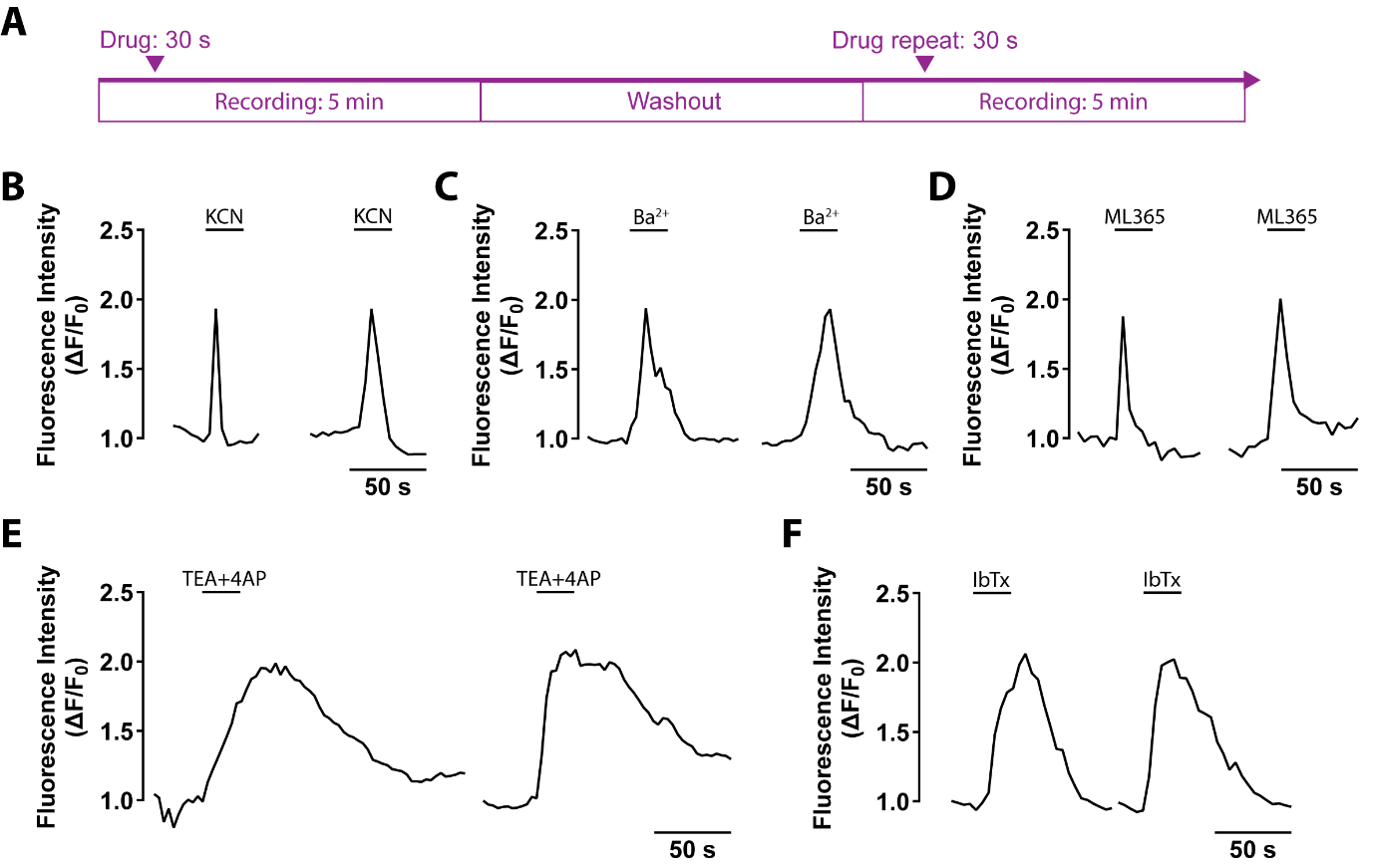


**Supplement Figure 4. Drug reversibility and reproducibility on glomus cells.** (A) Experimental protocol. Each drug repeat is tested on the same glomus cell with a 10 min washout in between. (B) Potassium cyanide (KCN, 1 mM); (C) Barium chloride (Ba^2+^, 10 mM); (D) ML365, 1µM; (E) Tetraethylammonium (TEA, 10 mM) and 4-aminopyridine (4AP, 5 mM); (F) Iberiotoxin (IbTx, 100 nM).


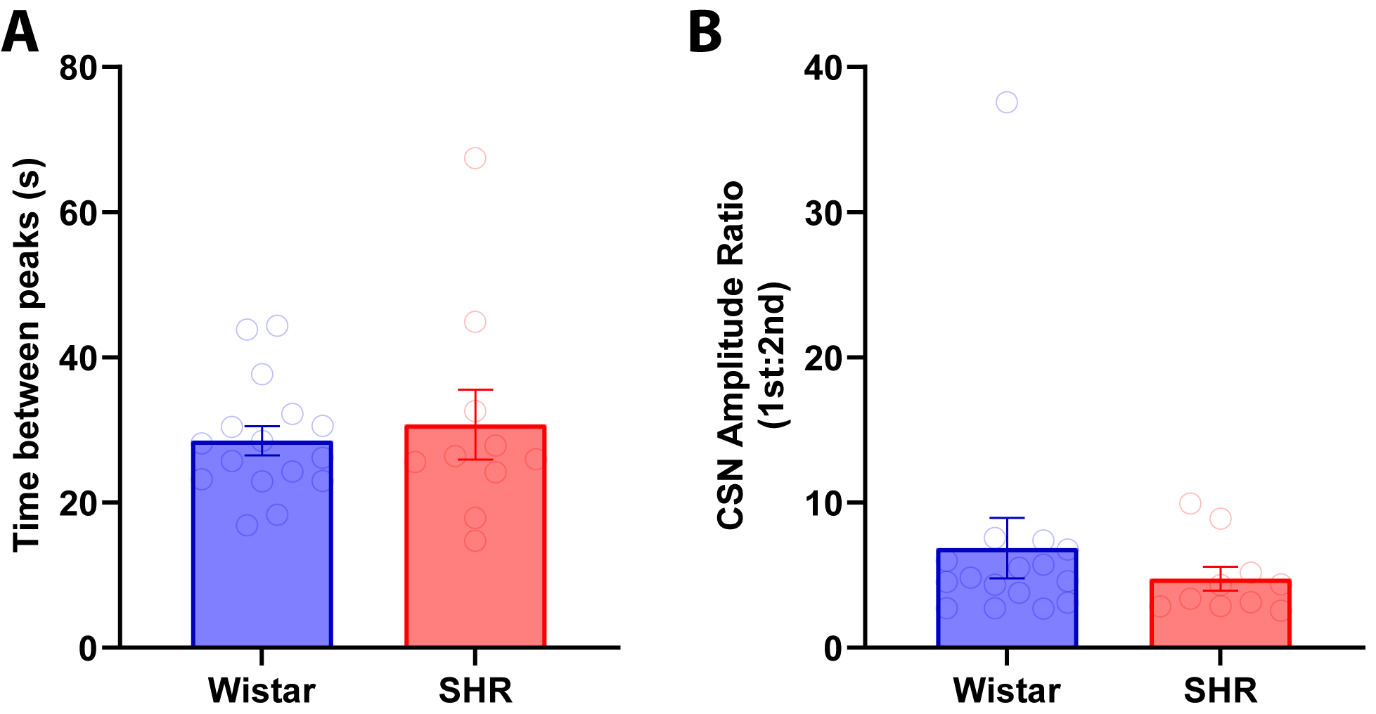


**Supplement Figure 5.** (A) Time difference between the 1st and the 2nd peak of the biphasic response in the Wistar and the SHR. (B) Response amplitude ratio (1st:2nd) between the Wistar and the SHR. For Wistar, n=16 animals; SHR, n=10 animals. p values calculated were using the Mann-Whitney U test.


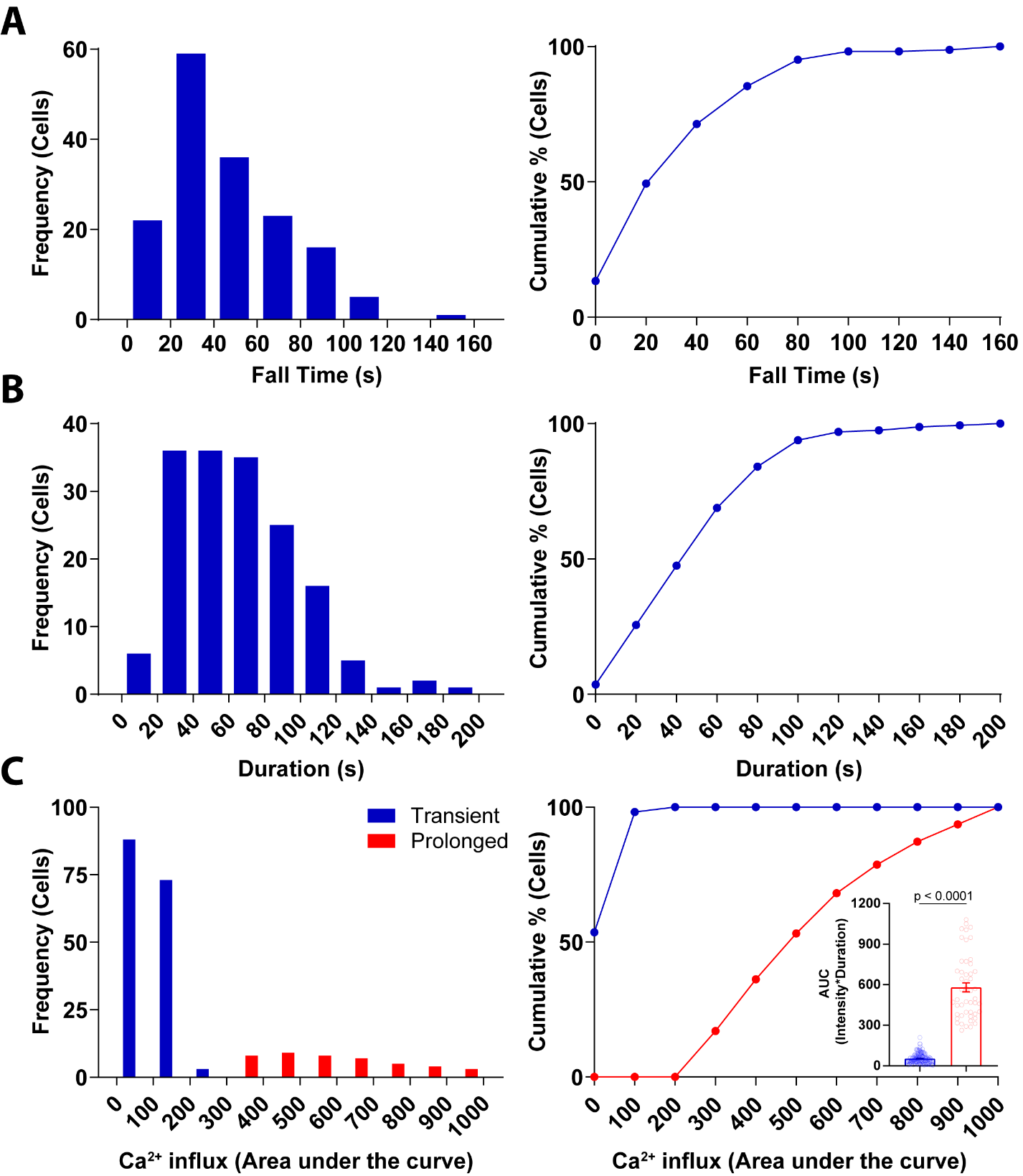


**Supplement Figure 6. KCN-mediated transient and prolonged Ca^2+^ event characteristics in Wistar CB glomus cells.** Frequency and cumulative frequency distribution of (A) Fall time; (B) Duration and (C) total Ca^2+^ influx for transient and prolonged Ca^2+^ events. n=213 cells/6 animals. Error bars are ± SEMs. p values calculated were using the Mann-Whitney U test.


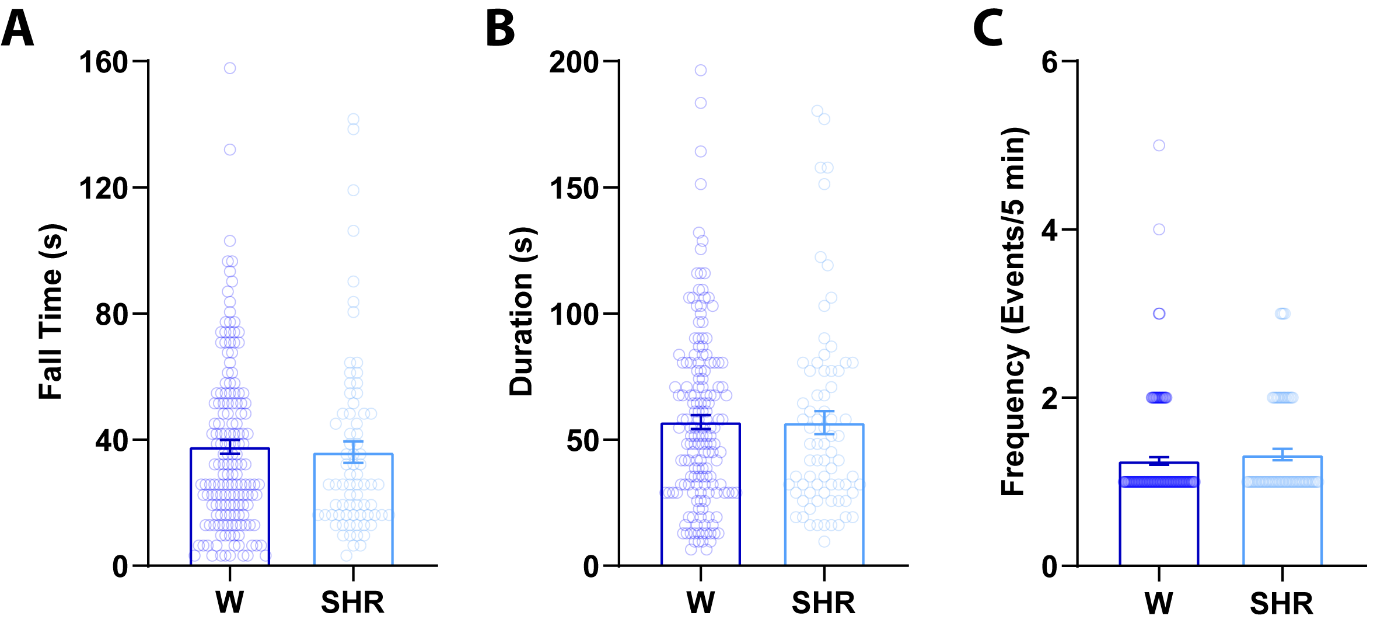


**Supplement Figure 7.** (A) Fall time, (B) duration and (C) frequency of transient Ca^2+^ events evoked by KCN between the Wistar and the SHR. For Wistar, n=213 cells/6 animals; for SHR, n=136/9 animals. Error bars are ± SEMs. p values were calculated using the Mann-Whitney U test.


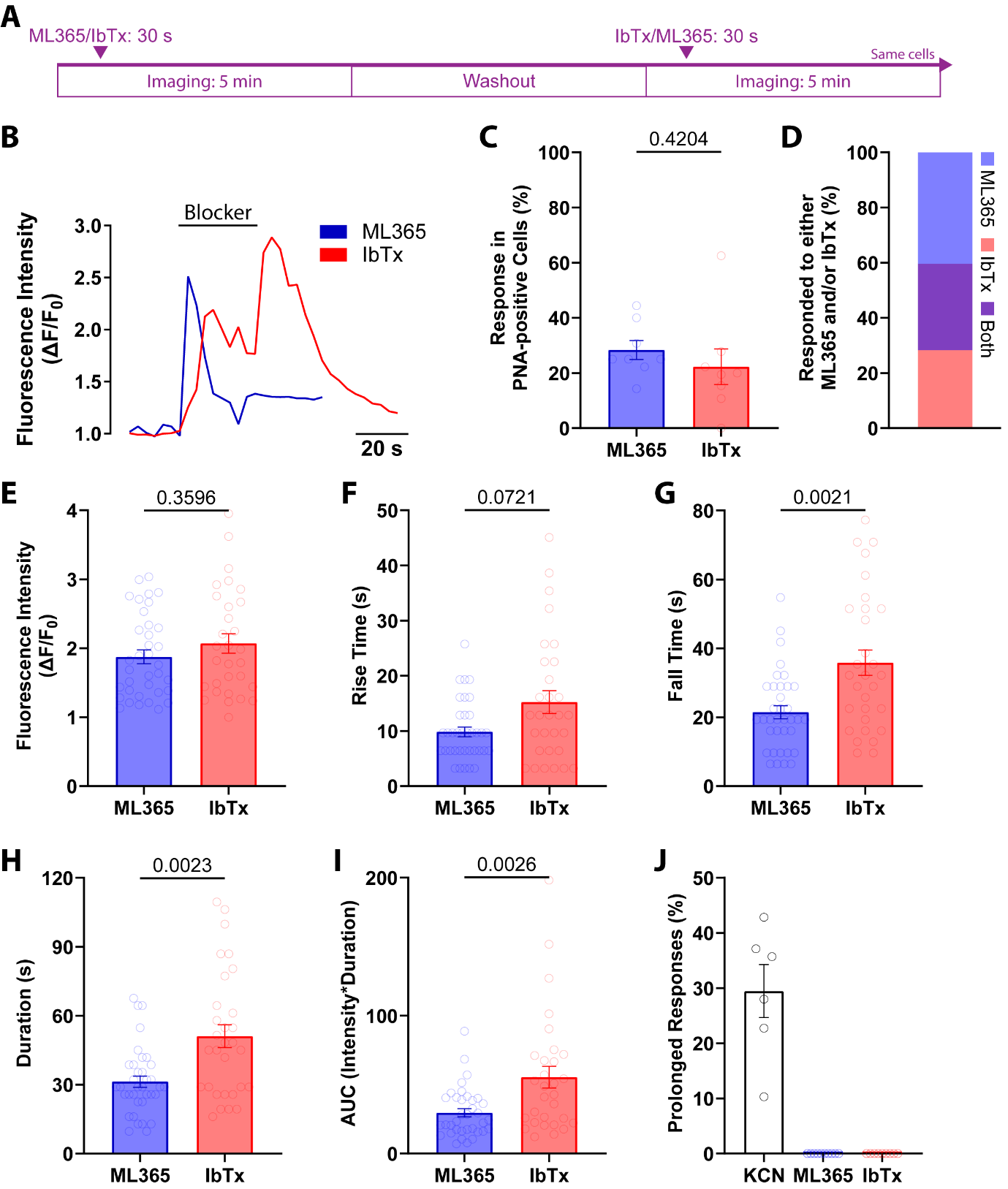


**Supplement Figure 8. Specific pharmacological inhibition of TASK and BK channels give rise to distinct Ca^2+^ events in Wistar CB glomus cells.** (A) Experimental protocol outline depicting a single imaging run. TASK channel inhibitor ML365 (1 µM) or BK channel inhibitor Iberiotoxin (IbTx, 0.1 µM) were given in randomised orders in each run. Each run was carried out on the same cells (B) Representative traces showing two distinct types of Ca^2+^ events evoked by the addition of either ML365 or IbTx in glomus cells. (C) ML365- or IbTx-sensitive cells as a percentage of all PNA-positive glomus cells. (D) Percentage of glomus cells that responded to ML365 and/or IbTx. (E-I) ML365- and IbTx-mediated Ca^2+^ event intensity, rise time, fall time, duration, and AUC (Ca^2+^ influx magnitude) in glomus cells. (J) Percentage of prolonged Ca^2+^ events evoked by KCN, ML365 or IbTx. n=50 cells/4 animals. Error bars are ± SEMs. p values were calculated using the Mann-Whitney U test.


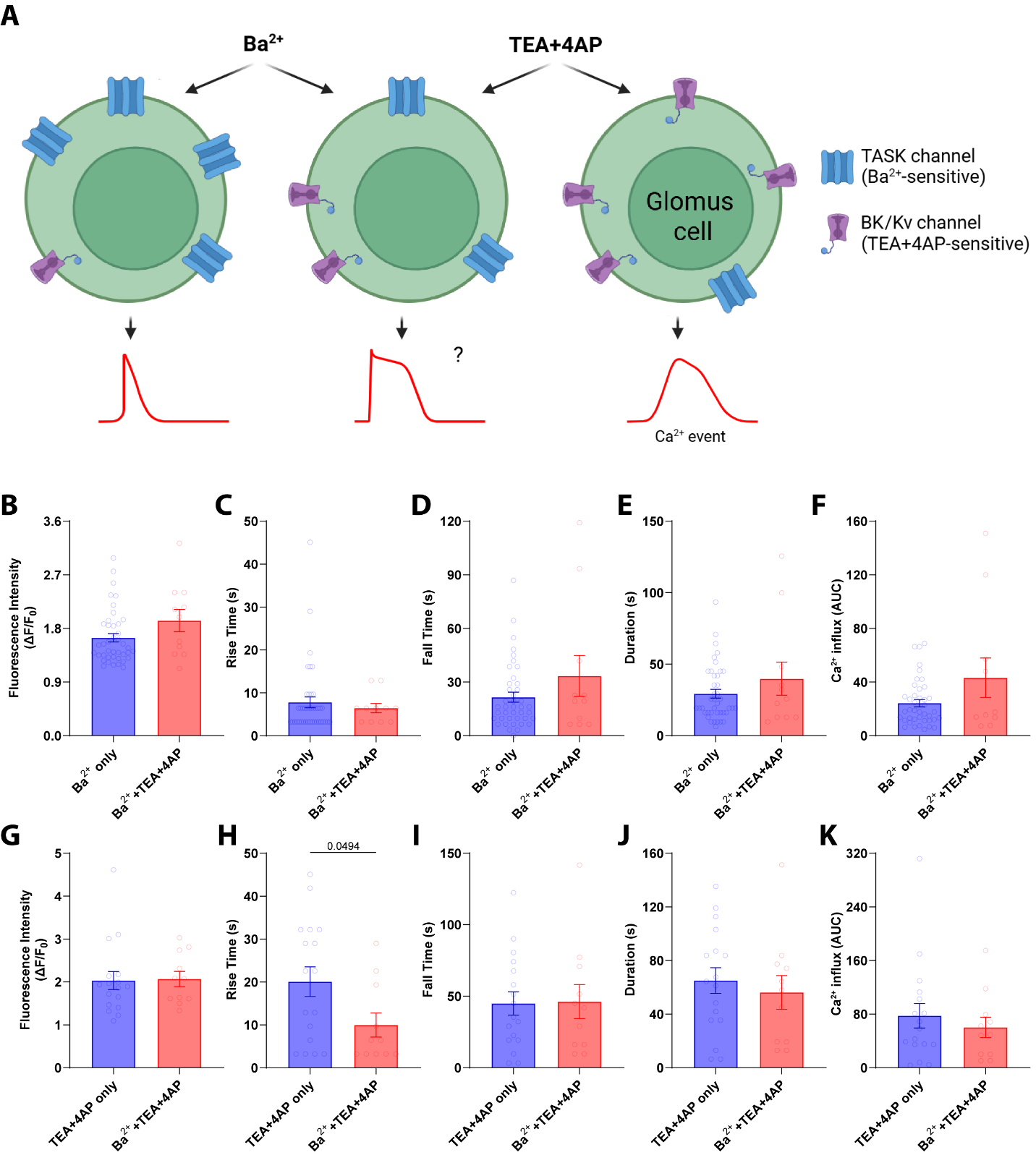


**Supplement Figure 9. Ca^2+^ events between subpopulations of glomus cells sensitive to Ba^2+^ only, TEA+4AP only and to both Ba^2+^ and TEA+4AP.** (A) Schematic illustration outlining the experimental protocol. (B) Fluorescent intensity; (C) Rise time; (D) Fall time; (E) Duration and (F) Ca^2+^ influx of Ca^2+^ events evoked by Ba^2+^ between glomus cells sensitive to Ba^2+^ only and those sensitive to both Ba^2+^ and TEA+4AP. (G) Fluorescent intensity; (H) Rise time; (I) Fall time; (J) Duration and (K) Ca^2+^ influx of Ca^2+^ events evoked by TEA+4AP between glomus cells sensitive only to TEA+4AP and those sensitive to both Ba^2+^ and TEA+4AP. n= 69 cells/5 animals. Error bars are ± SEMs. p values were calculated using the Mann-Whitney U test.


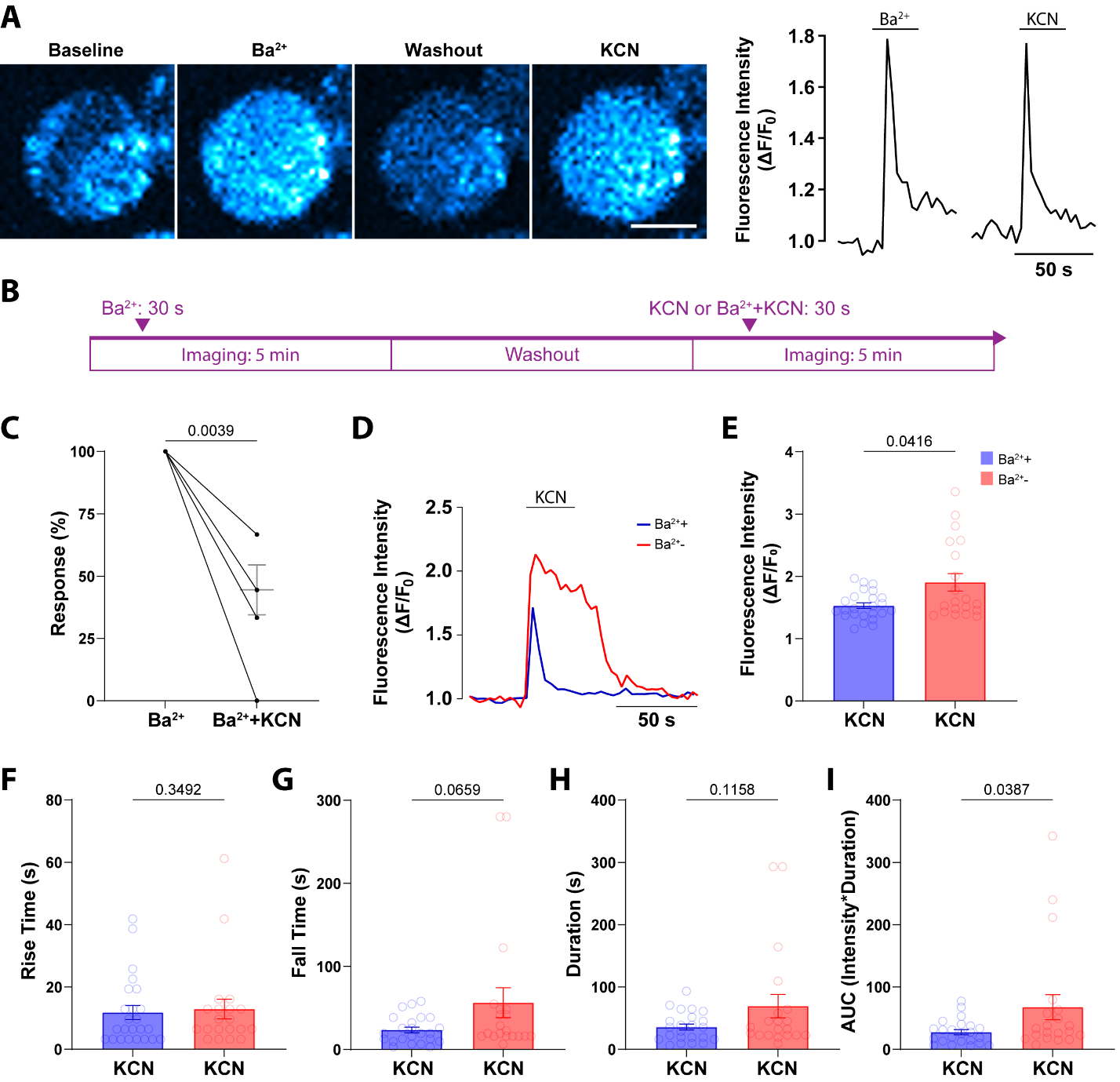


**Supplement Figure 10. Ba^2+^ partially inhibits KCN-sensitivity. KCN-mediated Ca^2+^ events in Ba^2+^-sensitive cells exhibit reduced fluorescence intensity and Ca^2+^ influx magnitude when compared to Ba^2+^-insensitive cells.** (A) Representative images and traces showing Fluo-4 loaded glomus cells that are sensitive to Ba^2+^ are also sensitive to KCN. Scale bar: 5 µm. (B) Experimental protocol outlining the initial identification of Ba^2+^-sensitive glomus cells. This was followed by either examining the effects of Ba^2+^ inhibition on KCN-sensitivity or comparing KCN-mediated Ca^2+^ events between Ba^2+^-sensitive (Ba^2+^+) and Ba^2+^-insensitive (Ba^2+^-) cells. (C) Percentage of Ba^2+^-sensitive glomus cells (also KCN-sensitive) which remained responsive to KCN in the presence of Ba^2+^ inhibition. (D) Representative trace comparing KCN-mediated Ca^2+^ events between Ba^2+^+ and Ba^2+^- cells. (E-I) KCN-mediated Ca^2+^ event intensity, rise time, fall time, duration and AUC (Ca^2+^ influx magnitude) between Ba^2+^+ and Ba^2+^- glomus cells. n=73 cells/4 animals. Error bars are ± SEMs. p values were calculated using Wilcoxon matched-paired signed rank test for (C) and Mann-Whitney U test for (E-I).


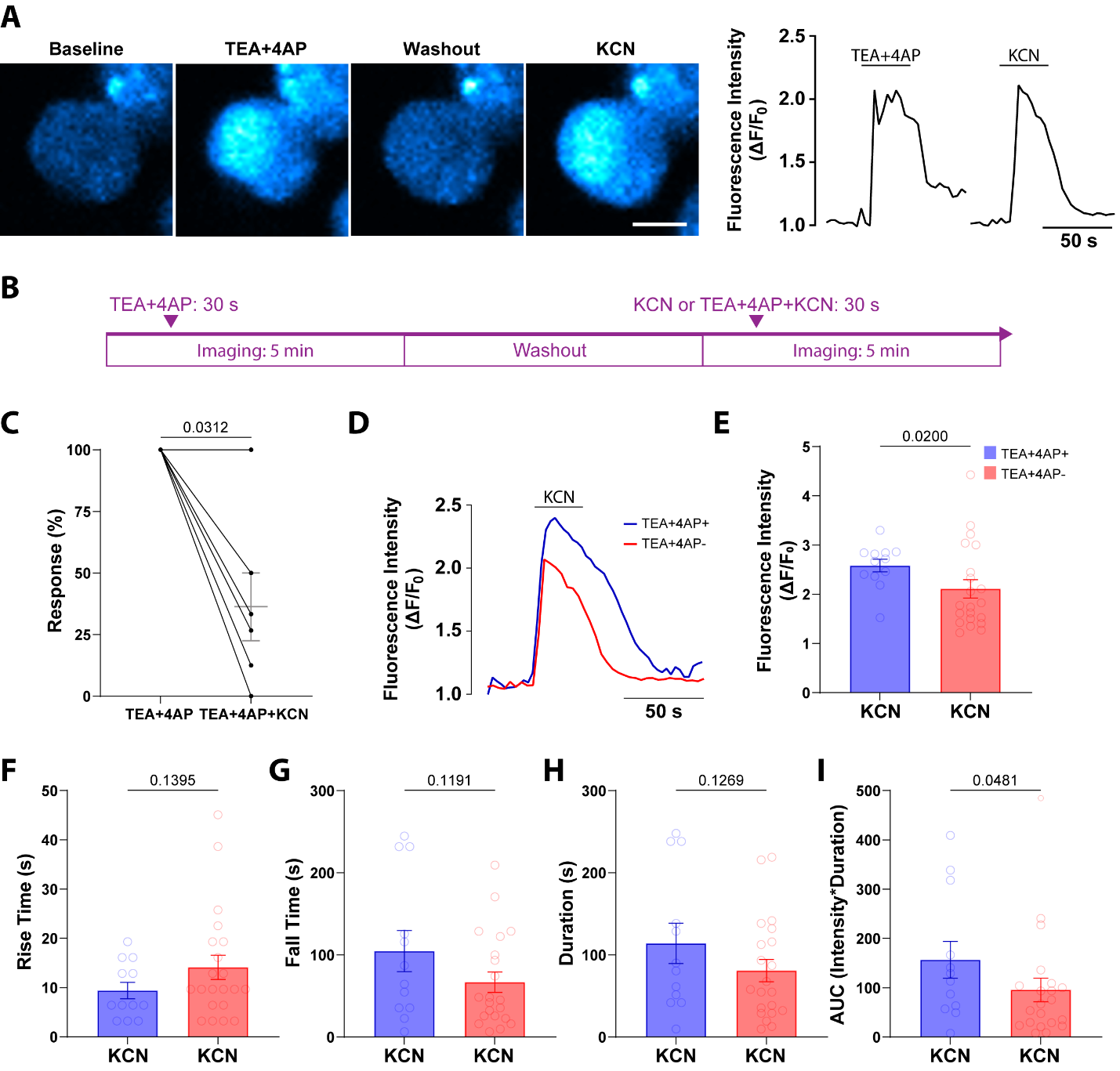


**Supplement Figure 11. TEA+4AP partially inhibits KCN-sensitivity. KCN-mediated Ca^2+^ events in TEA+4AP-sensitive cells exhibit greater fluorescence intensity and Ca^2+^ influx magnitude when compared to TEA+4AP-insensitive cells. (A)** Representative images and traces showing Fluo-4 loaded glomus cells that are sensitive to TEA+4AP are also sensitive to KCN. Scale bar: 5 µm. (B) Experimental protocol outlining the initial identification of TEA+4AP-sensitive glomus cells. This was followed by either examining the effects of TEA+4AP inhibition on KCN-sensitivity or comparing KCN-mediated Ca^2+^ events between TEA+4AP-sensitive (TEA+4AP+) and TEA+4AP-insensitive (TEA+4AP-) cells. (C) Percentage of TEA+4AP-sensitive glomus cells (also KCN-sensitive) which remained responsive to KCN in the presence of TEA+4AP inhibition. (D) Representative trace comparing KCN-mediated Ca^2+^ events between TEA+4AP+ and TEA+4AP- cells. (E-I) KCN-mediated Ca^2+^ event intensity, rise time, fall time, duration and AUC (Ca^2+^ influx magnitude) between TEA+4AP+ and TEA+4AP- glomus cells. n=58 cells/4 animals. Error bars are ± SEMs. p values were calculated using Wilcoxon matched-paired signed rank test for (C) and Mann-Whitney U test for (E-I).


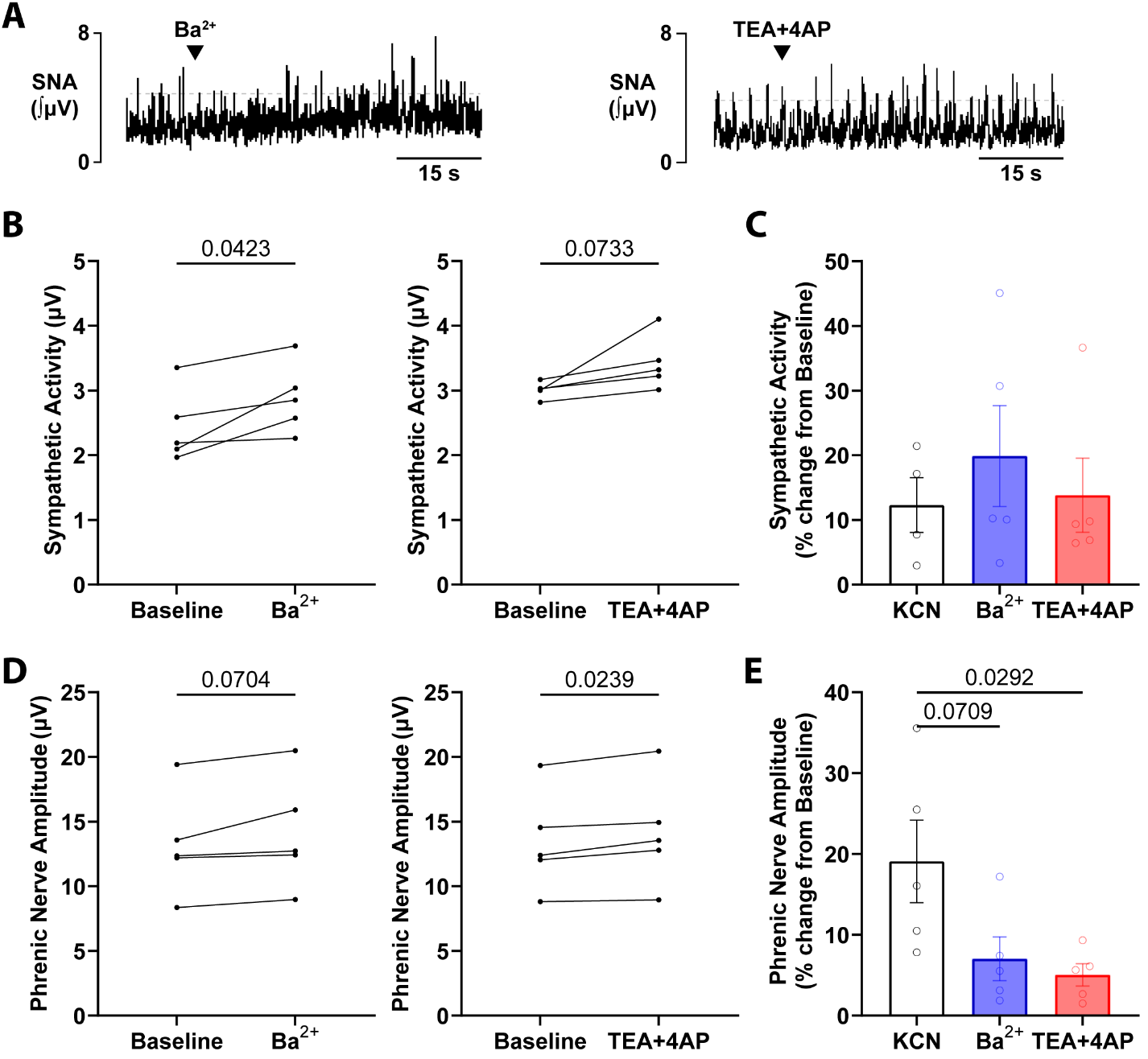


**Supplement Figure 12. Thoracic sympathetic nerve activity, phrenic nerve amplitude and perfusion pressure following Ba^2+^ and TEA+4AP inhibition in the Wistar rat dpWHBP.** (A) Representative traces showing changes in thoracic sympathetic nerve activity (SNA) after perfusing the CB with either Ba^2+^ or TEA+4AP. (B) Increase in thoracic sympathetic nerve activity above baseline after Ba^2+^ or TEA+4AP infusion. (C) Relative increase in thoracic sympathetic nerve activity from baseline induced by KCN, Ba^2+^, or TEA+4AP. (D) Increase in phrenic nerve amplitude above baseline after Ba^2+^ or TEA+4AP infusion. (E) Relative increase in phrenic nerve amplitude from baseline induced by KCN, Ba^2+^, or TEA+4AP. n= 5 animals. Error bars are ± SEMs. p values were calculated using paired t-test.


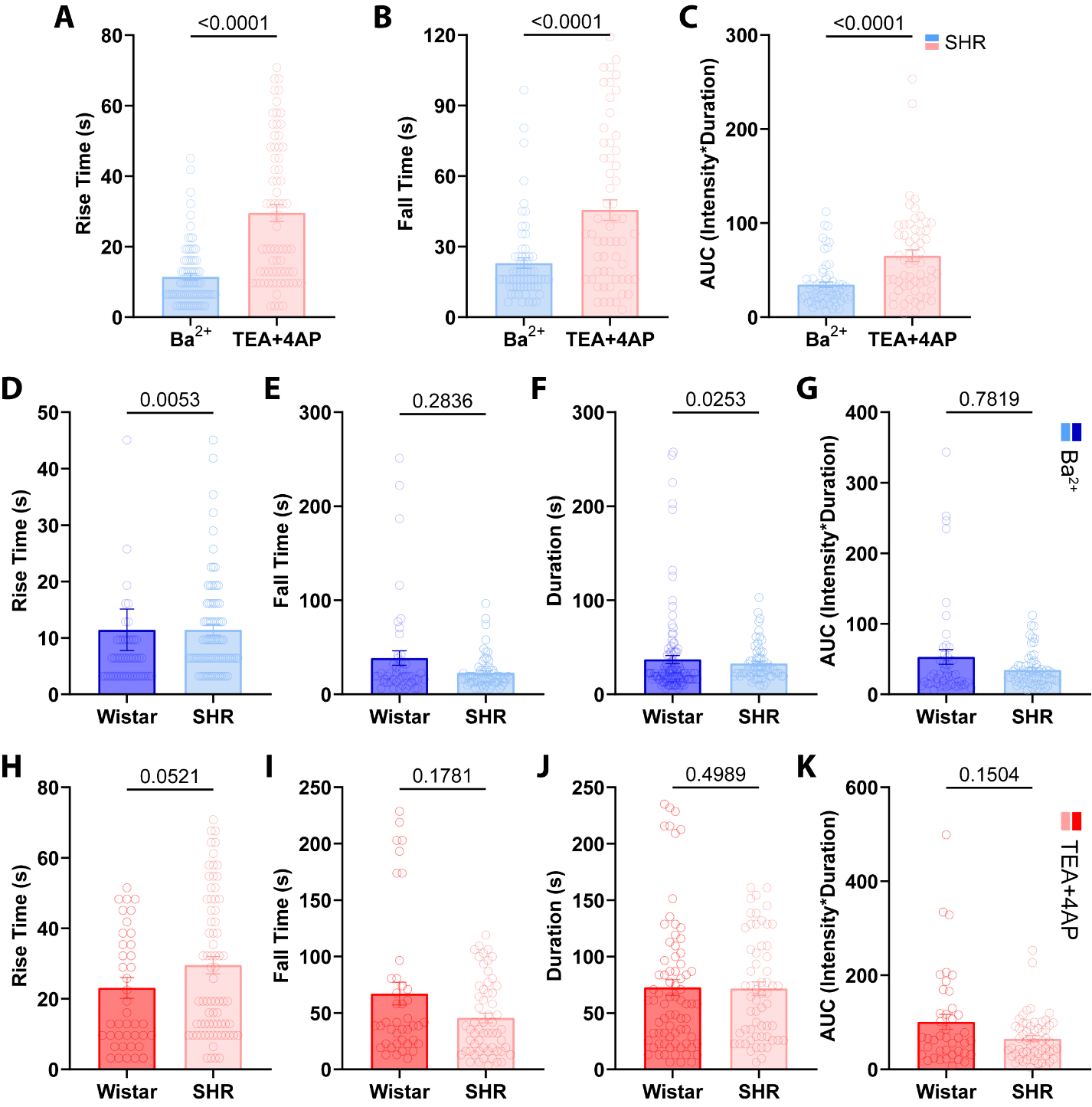


**Supplement Figure 13. Distinct Ba^2+^- and TEA+4AP-evoked Ca^2+^ events in SHRs compared with Wistar rats.** (A-C) Rise time, fall time and AUC (Ca^2+^ influx magnitude) in Ca^2+^ events evoked by Ba^2+^ or TEA+4AP in glomus cells from SHRs. (D-G) Rise time, fall time, duration and AUC of Ba^2+^-evoked transient Ca^2+^ events in glomus cells from Wistar and SHR rats. (H-K) Rise time, fall time, duration and AUC of TEA+4AP-evoked transient Ca^2+^ events in glomus cells from Wistar and SHR rats. For Wistar, n=231 cells/5 animals; for SHR, n=125 cells/4 animals. Error bars are ± SEMs. p values were calculated using the Mann-Whitney U test.


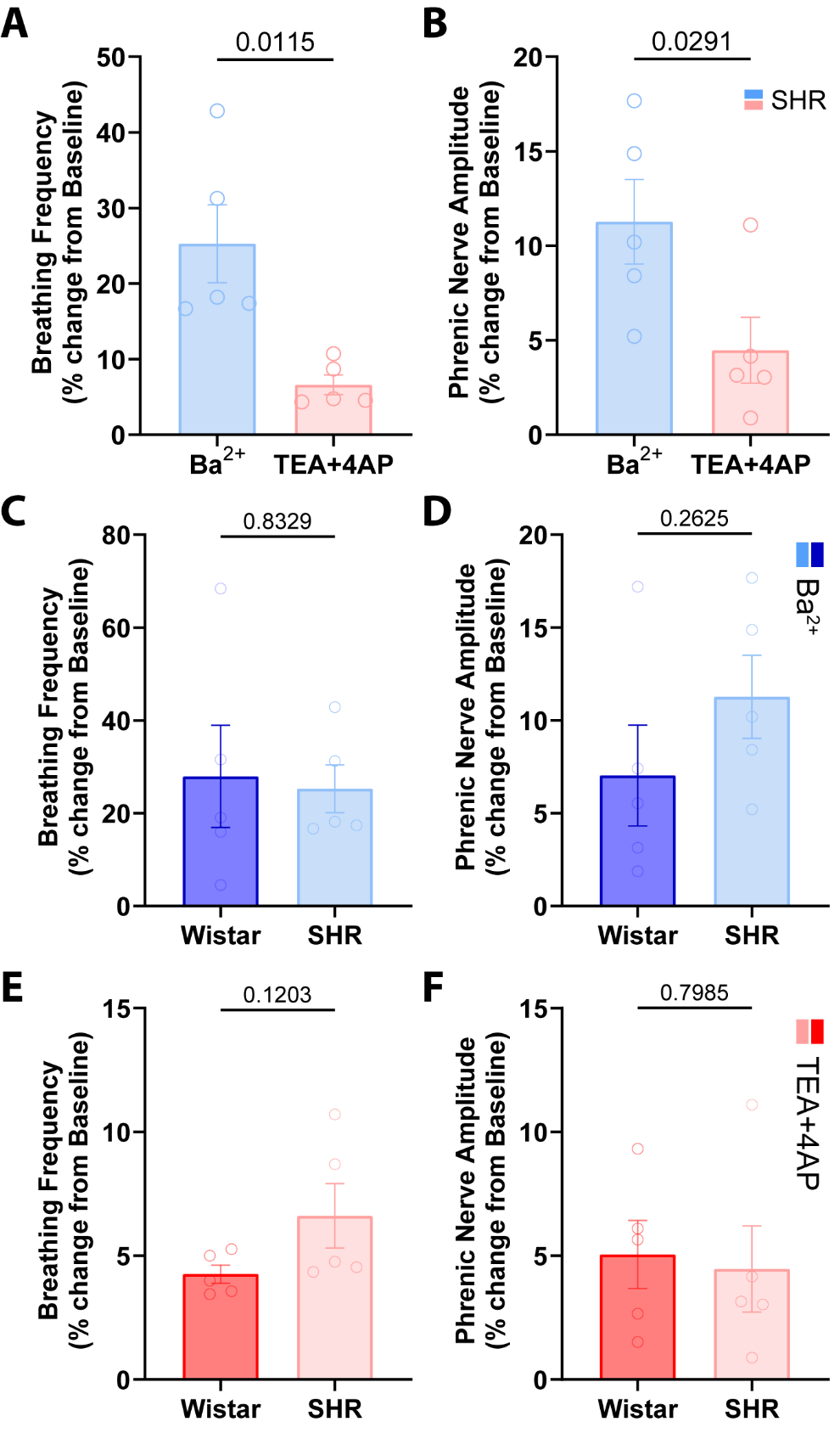


**Supplement Figure 14. Distinct Ba^2+^- and TEA+4AP-evoked chemoreflex responses in SHRs compared with Wistar rats. (A, B)** Relative changes from baseline in breathing frequency and phrenic nerve amplitude induced by Ba^2+^ or TEA+4AP in SHRs. (C, D) Relative changes from baseline in breathing frequency and phrenic nerve amplitude induced by Ba^2+^ between Wistar rats and SHRs. (E, F) Relative changes from baseline in breathing frequency and phrenic nerve amplitude induced by TEA+4AP between Wistar rats and SHRs. For Wistar, n=5 animals; for SHR, n=5 animals. Error bars are ± SEMs. p values were calculated using unpaired t-test.


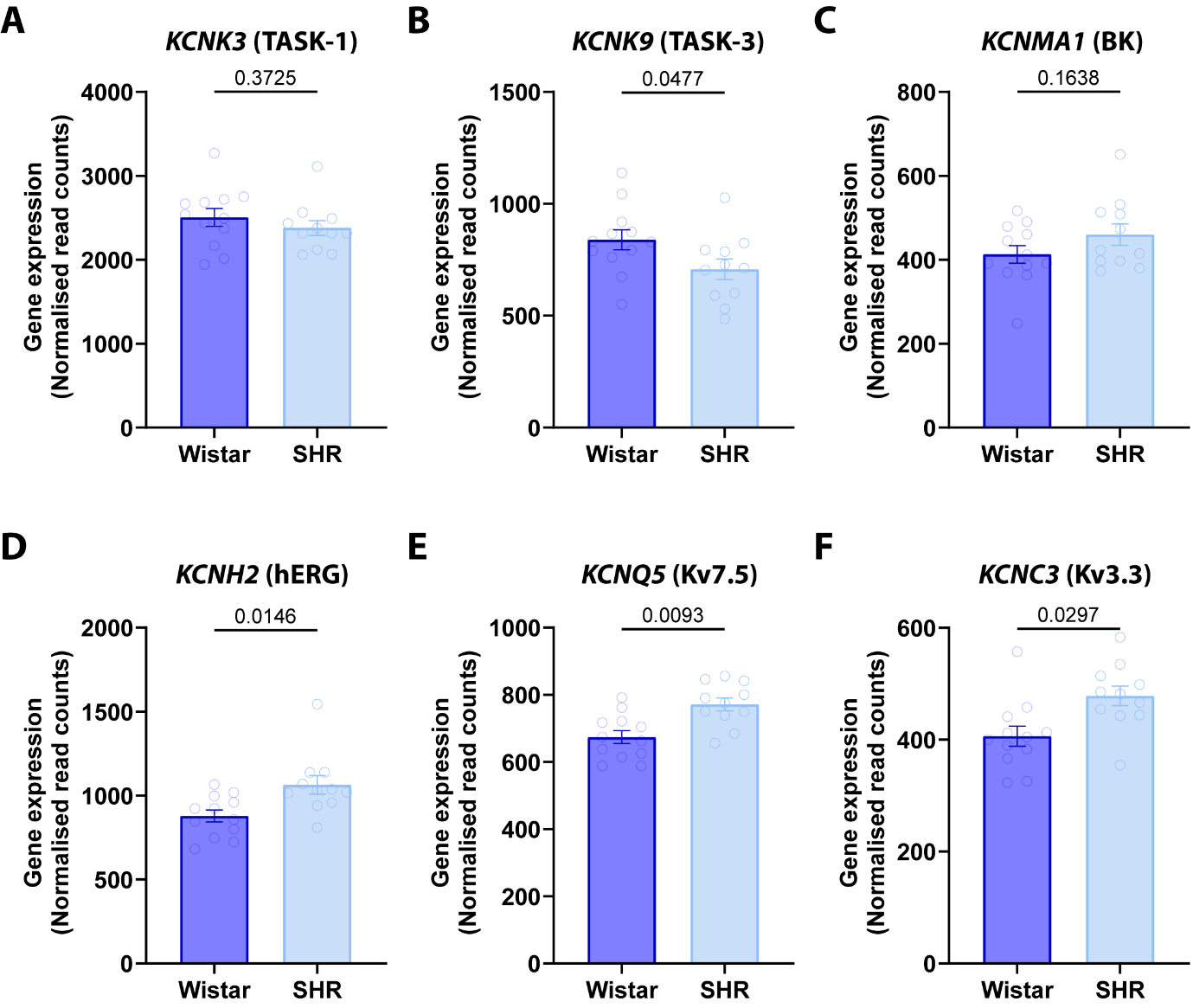


**Supplement Figure 15. Key K^+^ channels exhibit differential expression in the CB of Wistars and SHRs.** K^+^ channels gene expression was detected by RNA-seq, as published originally^4^. (A-F) Gene expression comparison of *KCNK3*, *KCNK9*, *KCNMA1*, *KCNH2*, *KCNQ5* and *KCNC3* between Wistars and SHRs. For Wistars, n=12 animals; for SHR, n=11 animals. Error bars are ± SEMs. P values were calculated using unpaired t-test. Benjamini-Hochberg correction (pAdj < 0.05) was used for comparison.


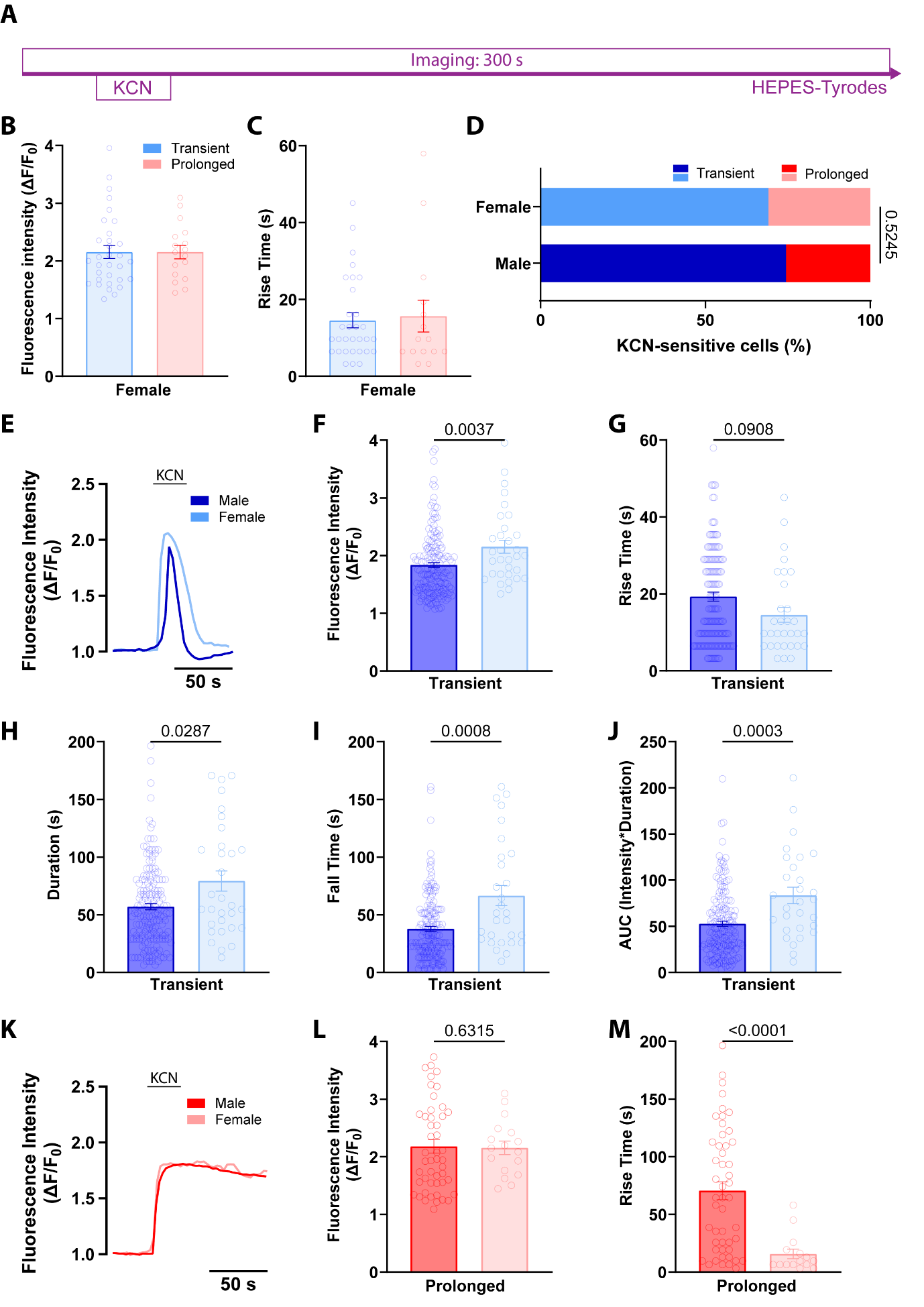


**Supplement Figure 16. Sex differences in KCN-mediated Ca^2+^ events in glomus cells from Wistar rats.** (A) Experimental protocol. (B, C) Comparison of transient and prolonged Ca^2+^ event intensity and rise time in glomus cells from female Wistar rats. (D) Relative distribution of transient and prolonged Ca^2+^ events in KCN-sensitive glomus cells from male and female Wistar rats. (E) Representative traces of transient Ca^2+^ events in glomus cells from male and female Wistar rats. (F-J) Comparison of Ca^2+^ event intensity, rise time, fall time, duration, and magnitude (AUC) between glomus cells from male and female Wistar rats. (K) Representative traces of prolonged Ca^2+^ events in glomus cells from male and female Wistar rats. (L, M) Comparison of Ca^2+^ event intensity and rise time between glomus cells from male and female Wistar rats. For males, n=213 cells/6 animals; for females, n=46 cells/3 animals. Error bars are ± SEMs. p values calculated were using the Mann-Whitney U test.

**References**

1. Pauza AG, Thakkar P, Shen X, Felippe I, Roßmann K, Oya M, et al. Melanocortin system activates carotid body arterial chemoreceptors in hypertension. bioRxiv : the preprint server for biology. 2024:2024.07.22.604704.

2. Paton JF. A working heart-brainstem preparation of the mouse. Journal of neuroscience methods. 1996;65(1):63-8.

3. Faul F, Erdfelder E, Buchner A, Lang A-G. Statistical power analyses using G*Power 3.1: Tests for correlation and regression analyses. Behavior Research Methods. 2009;41(4):1149-60.

4. Pauza AG, Thakkar P, Tasic T, Felippe I, Bishop P, Greenwood MP, et al. GLP1R Attenuates Sympathetic Response to High Glucose via Carotid Body Inhibition. Circulation research. 2022;130(5):694-707.
